# gwSPADE: gene frequency-weighted reference-free deconvolution in spatial transcriptomics

**DOI:** 10.1093/nar/gkaf966

**Published:** 2025-09-26

**Authors:** Aoqi Xie, Nina G Steele, Yuehua Cui

**Affiliations:** Department of Statistics and Probability, Michigan State University, East Lansing, MI 48824, United States; Department of Surgery, Henry Ford Michigan State University Pancreatic Cancer Center, 4-Henry Ford Health, Detroit, MI 48202, United States; Department of Pharmacology and Toxicology, Michigan State University, East Lansing, MI 48824, United States; Department of Oncology, Wayne State University, Detroit, MI 48201, United States; Division of Gastroenterology and Hepatology, Department of Internal Medicine, College of Medicine, University of Cincinnati, Cincinnati, OH 45267, United States; Department of Statistics and Probability, Michigan State University, East Lansing, MI 48824, United States

## Abstract

Most spatial transcriptomics (ST) technologies (e.g. 10× Visium) operate at the multicellular level, where each spatial location often contains a mixture of cells with heterogeneous cell types. Thus, effective deconvolution of cell type compositions is critical for downstream analysis. Although reference-based deconvolution methods have been proposed, they depend on the availability of reference data, which may not always be accessible. Additionally, within a deconvolved cell type, cellular heterogeneity may still exist, requiring further deconvolution to uncover finer structures for a better understanding of this complexity. Here, we present gwSPADE, a **g**ene frequency-**w**eighted reference-free **SPA**tial **DE**convolution method for ST data. gwSPADE requires only the gene count matrix and utilizes appropriate weighting schemes within a topic model to accurately recover cell type transcriptional profiles and their proportions at each spatial location, without relying on external single-cell reference information. In various simulations and real data analyses, gwSPADE demonstrates scalability across various platforms and shows superior performance over existing reference-free deconvolution methods such as STdeconvolve.

## Graphical Abstract

**Graphical Abstract:**
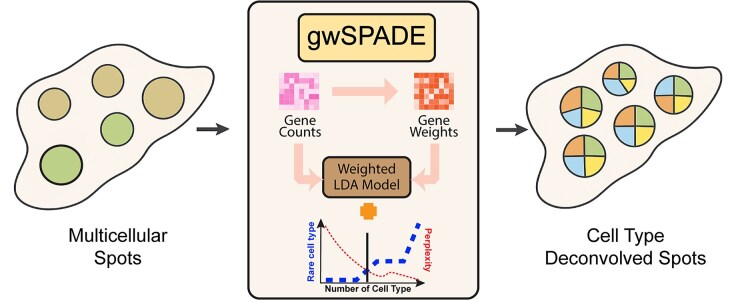

## Introduction

Understanding gene expression is pivotal to deciphering tissue functionality [1, 2], which prompts rapid development of various high-throughput sequencing technologies aimed at capturing detailed gene expressions. Though recent methods like MERFISH [3] enable the acquisition of data at single-cell resolution, for broad commercial use, technologies such as spatial transcriptomics (ST) in spot resolution, including 10× Visium [4], offer a good compromise between in-depth molecular detail and scalability. However, a primary limitation of such ST technologies is that the gene expression data captured at a single location (such as a spot or grid) is typically a composition of multiple cells. This blending makes it challenging to accurately pinpoint spatial patterns specific to individual cell types or detect subtle gene expression differences. To address the challenge of accurately identifying cell types within spatial locations, a variety of deconvolution methods have been developed [5, 6], and they can be broadly categorized into reference-based and reference-free approaches.

Reference-based deconvolution methods, such as RCTD [7], Stereoscope [8], Cell2location [9], SPOTlight [10], and CARD [11], rely on external datasets to guide the deconvolution process. These methods employ statistical models ranging from Poisson and negative binomial regression to more advanced frameworks like nonnegative matrix factorization (NMF) and conditional autoregressive modeling. By leveraging existing scRNA-seq data, reference-based methods excel at accurately identifying known cell types. However, their effectiveness is restricted by the quality and availability of reference datasets, which may not fully capture the diversity of cell types in unknown or highly heterogeneous tissues [12, 13]. In some applications, batch effects across different platforms pose a significant challenge to their reliability [14, 15]. Furthermore, these methods might not be well-suited for uncovering novel cell types or capturing dynamic changes in gene expression patterns that are not represented in the reference data. Additionally, after reference-based deconvolution, researchers are often interested in uncovering sub-cell types within regions dominated by major cell types, especially when reference profiles for those subtypes are unavailable. These limitations motivated the development of reference-free methods, such as SpiceMix [16] and STdeconvolve [17], which do not rely on external datasets. Ref-free methods infer cell types directly from ST data, enabling the discovery of novel cell types or sub-cell types and providing insights into tissues with poorly characterized cell populations.

SpiceMix builds upon NMF model, incorporating spatial graph information by applying hidden Markov random fields (HMRF) to deconvolve each spot as a mixture of latent factors (metagenes); it is not specifically tailored for spatial cell type deconvolution [18] and lacks a built-in criterion for selecting the optimal number of deconvolved cell types [16]. Consequently, it often fails to accurately distinguish distinct cell type patterns in real-world data [5].

As one of the well-received ref-free methods, STdeconvolve [17] leverages Latent Dirichlet Allocation (LDA) model [19], a generative probabilistic model that facilitates the discovery of cell types within ST data. The LDA model, known as a topic model, was developed for document classification. It treats documents as mixtures of latent topics, each with a unique frequency profile of word usage. Similarly, in ST data, each multi-cellular spatial location is analogous to a document, and cell types within that location correspond to hidden topics. RNA sequencing data align closely with LDA concepts with gene transcripts measured as counts, making the model suitable for this application. Table 1 provides an analogy between terms used in LDA modeling and ST.

**Table 1.** Analogy between natural language processing and spatial transcriptomics

Natural Language Processing	Spatial Data Analog
Corpus	The entire set of ST spots or cells
Document	A spatial spot, capture location, or cell
Word	A gene
Vocabulary	The set of all genes measured
Topic	A latent cell type

STdeconvolve, while using the standard LDA model effectively, faces challenges in distinguishing cell types due to the unequal contribution of genes. In deconvolved cell type-specific transcriptional profiles, the most frequent genes typically include expected marker genes used for identification. However, highly expressed genes that appear across the entire dataset can dominate certain cell types, leading to their presence in multiple topics without clear significance [20], making them less informative. Beyond the composition proportions of deconvolved cell types, the gene expression profiles within each cell type are crucial for downstream analyses, such as gene set enrichment analysis. Therefore, developing methods that account for the influence of highly expressed genes is essential to prevent them from disproportionately affecting multiple cell types, which could lead to biologically meaningless results.

In natural language processing, term weighting is recognized as essential for addressing imbalances caused by high-frequency words across the dataset [20–25]. Similarly, in ST data, effective weighting methods are crucial to down-weight high-frequency genes and improve the weight of discriminative genes. This approach ensures that specific genes stand out in particular cell types, thereby reducing the disproportionate influence of certain genes across multiple cell types. Consequently, this improves cell type identification and enhances accuracy in understanding the composition of spatial spots.

When applying STdeconvolve to a real pancreatic ST dataset, we observed that some highly expressed genes appeared as top genes across multiple cell types, resulting in indiscriminate deconvolution. Meanwhile, certain cell type-specific marker genes did not exhibit high expression. Therefore, down-weighting these high-frequency genes while up-weighting informative low-frequency genes could lead to enhanced deconvolution precision. Motivated by this observation and considering the limitation of STdeconvolve, here we developed a **g**ene expression-**w**eighted reference-free **SPA**tial **DE**convolution (gwSPADE) method for multicellular ST deconvolution. gwSPADE extends the traditional LDA model by incorporating term weighting schemes, which significantly enhance the accuracy of predicting cell type proportions within spots by better capturing cell type gene profile heterogeneity. We comprehensively evaluated the performance of different weighting schemes. The result showed the superior performance of gwSPADE equipped with the balanced distributional concentration (BDC) weighting scheme across four published ST datasets, each characterized by different technologies, spatial resolutions, and tissue architectures. The advantages of gwSPADE-BDC were further demonstrated through applications to one model-based simulation and two single-cell-derived simulated ST datasets.

## Materials and methods

### Overview of gwSPADE

**Figure 1.**
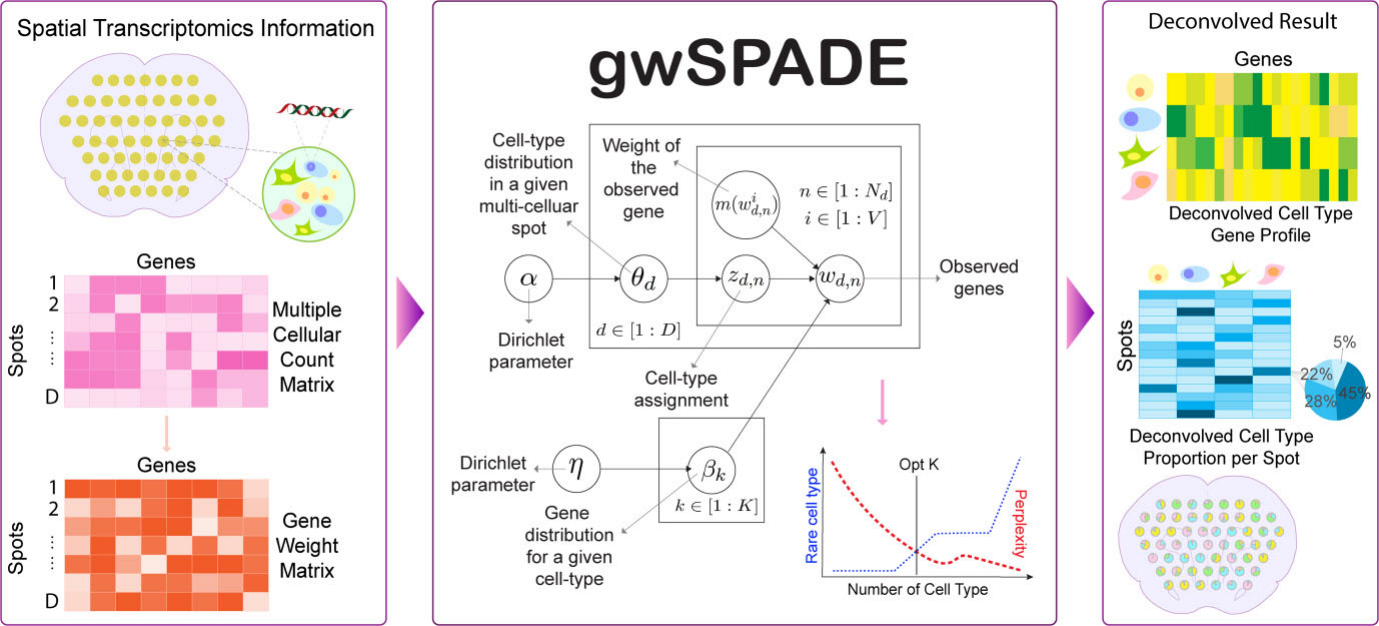
Overview of gwSPADE. gwSPADE takes as input a ST count matrix, where rows correspond to spots and columns correspond to genes. Within the gwSPADE model, it constructs a weight matrix of the same dimensions, assigning weights to each gene in every spot. Both the weighted and original count matrices are then utilized in an unsupervised topic model to generate two primary outputs: the cell type transcriptional profile, captured as a cell type-by-gene matrix, and the cell type composition, represented as a spot-by-cell type matrix. Detailed descriptions of the model’s variables and methods are provided in the “Materials and methods” section.

gwSPADE model is an improved method based on the regular LDA model [19], a generative probabilistic model, to discern latent cell types within multi-cellular ST data. In the context of multicellular ST, each spatial location (i.e., spot) *d* ∈ [1, ..., *D*] is conceptualized as a composite of *K* distinct cell types where *D* is the total number of spots for a tissue. These cell types can be mathematically represented through a multinomial distribution of cell type probabilities θ_*d*_ in each spot, with each cell type *k* ∈ [1, ..., *K*] further characterized by a probability distribution β_*k*_ over *V* genes. Figure 1 shows the main framework of gwSPADE. Before we introduce the model, we first define the following notations:

- θ = (θ_1_, ⋅⋅⋅, θ_*D*_)^*T*^ is a *D* × *K* matrix, where each row θ_*d*_ follows a uniform Dirichlet distribution with scalar parameter α, i.e. θ_*d*_ ∼ Dir(α), $\sum _k^K\theta _{d,k}=1$ ∀*d*, describing the composition of each cell type within spot *d*.
- β = (β_1_, ⋅⋅⋅, β_*K*_)^*T*^ is a *K* × *V* matrix, where each row β_*k*_ represents the relative gene expression frequency conditional on cell type *k*. This can be interpreted as the probability that gene *g* is expressed within cell type *k* in a Multinomial distribution. For each cell type *k*, the sum of the probabilities across all genes equals 1, i.e., $\sum _g^V \beta _{k,g} = 1 \ \text{for all} \ k$.
- *z*_*d*, *n*_ denotes the cell type label that the *n*th observed UMI (unique molecular identifier) in spot *d* belongs to; *n* ∈ [1, ..., *N*_*d*_] where *N*_*d*_ is the total UMI read counts in spot *d*; and *z*_*d*, *n*_ ∈ [1, ⋅⋅⋅, *K*] ∼ Multinom(θ_*d*_).
- *w*_*d*, *n*_ denotes the specific gene that the *n*th observed UMI in spot *d* comes from, conditional on the cell type assignment *z*_*d*, *n*_ obtained above. The gene can be drawn as $w_{d,n} \in \left[1, \cdots , V\right] \sim \text{Multinom}(\boldsymbol{\beta }_{z_{d,n}})$

Table 2 summarizes the notations and their biological interpretations.

**Table 2.** Summary of notations and their interpretations

Notations	Biological Interpretations
*D*	Number of total spots, spot *d* ∈ [1, ..., *D*]
*K*	Number of deconvolved cell types, cell type *k* ∈ [1, ..., *K*]
*V*	Number of selected genes, gene *g* ∈ [1, ..., *V*]
θ_*d*_	Cell type proportion in spot *d*
θ	Cell type proportion matrix across all spots
β_*k*_	Gene expression frequency in cell type *k*
β	Gene expression frequency matrix across all cell types
*z*_*d*, *n*_	Cell type label for the *n*th UMI in spot *d*
*z*	The collection of assigned cell type labels for all UMIs
*w*_*d*, *n*_	Specific gene that the *n*th UMI in spot *d* comes from
*w*	The collection of gene assignments for all UMIs
*N*_*gdk*_	Number of transcript molecules of gene *g* in spot *d* assigned to topic *k*
$N_{gdk}^{-(d,n)}$	Number of transcript molecules of gene *g* in spot *d* assigned to topic *k*, excluding *w*_*d*, *n*_

To identify the latent parameters θ and *z*, we solve the posterior distribution of the latent variables based on the observed gene expression data, i.e.


(1) \begin{eqnarray*}
p(\theta ,z|w,\alpha ,\beta ) = \frac{p(\theta ,z,w|\alpha ,\beta )}{p(w|\alpha ,\beta )} = \prod _{d=1}^Dp(\theta _d,z|w,\alpha ,\beta ).
\end{eqnarray*}

To estimate the latent parameters, we use the regular LDA model with a Collapsed Gibbs Sampler [26, 27]. Assuming that, for each cell type *k*, the probability of each gene β_*k*_ follows a uniform Dirichlet distribution with scaling parameter η, i.e. β_*k*_ ∼ Dir(η), then the posterior distribution can be written as:


(2) \begin{eqnarray*}
p(\theta ,z,\beta |w,\alpha ,\eta )=\frac{p(\theta ,z,w,\beta |\alpha ,\eta )}{p(w|\alpha ,\eta )}
\end{eqnarray*}

using default settings of η = 0.01 and α = 1/*K*. Finally, given the current state of *z*, the conditional probability of *z*_*d*, *n*_ is given as


(3) \begin{eqnarray*}
p(z_{d,n}=k|z_{-(d,n)},w)=\left(\frac{N_{\left(\cdot \right)dk}^{-(d,n)}+\alpha }{N_{\left(\cdot \right)d\left(\cdot \right)}+K\alpha }\right)\left(\frac{N_{g\left(\cdot \right)k}^{-(d,n)}+\eta }{N_{\left(\cdot \right)\left(\cdot \right)k}^{-(d,n)}+V\eta }\right) \nonumber\\
\end{eqnarray*}

where,

- *N*_*gdk*_: Number of transcript molecules of gene *g* in spot *d* assigned to topic *k*.
-
$N_{gdk}^{-(d,n)}$ : Number of transcript molecules of gene *g* in spot *d* assigned to topic *k*, excluding *w*_*d*, *n*_.

During each iteration of the Gibbs sampling, we sample a new value for *z*_*d*, *n*_ corresponding to each transcript molecular *w*_*d*, *n*_. After running the sampler for a burn-in period of several thousands of iterations, we estimate θ_*d*_ and β_*k*_ from the latent variable *z* as follows:


(4) \begin{eqnarray*}
\theta _{d,k}=\frac{N_{\left(\cdot \right)dk}+\alpha }{N_{\left(\cdot \right)d\left(\cdot \right)}+K\alpha }
\end{eqnarray*}

(5) \begin{eqnarray*}
\beta _{k,g}=\frac{N_{g\left(\cdot \right)k}+\eta }{N_{\left(\cdot \right)\left(\cdot \right)k}+V\eta }.
\end{eqnarray*}

In ST data, high-frequency genes are often distributed across numerous cell types without discernible significance. For instance, housekeeping genes could frequently appear across all cells, overshadowing more cell type-specific genes. Thus, even when marker genes are accessible for some cell types, they can still be indiscriminate. Ideally, we desire that the aggregation of high-frequency genes within each cell type coalesces into a coherent, meaningful, and discriminative cell type.

To briefly explain the intuition behind the gwSPADE model, consider generating gene expression from the gene distribution of a cell type. The probability of gene expression should be proportional to both its expression frequency and the gene weight $m(w_{d,n}^{g})$, where $w_{d,n}^{g}$ denotes that the *n*th gene in spot *d* is of gene type *g*. In STdeconvolve, each gene is assigned a uniform weight. In our proposed gwSPADE model, the gene weights vary according to their expressions. With appropriate gene weighting, high-frequency genes can be naturally assigned to a specific cell type rather than being scattered across many cell types. We incorporate the weighting term $m(w_{d,n}^{g})$ into equation (3) by replacing the counts denoted by *N* with weighted counts denoted by *M*, i.e.


(6) \begin{eqnarray*}
&& p(z_{d,n}=k|z_{-(d,n)},w) = \left(\frac{M_{\left(\cdot \right)dk}^{-(d,n)}+\alpha }{M_{\left(\cdot \right)d\left(\cdot \right)}+K\alpha }\right)\left(\frac{M_{g\left(\cdot \right)k}^{-(d,n)}+\eta }{M_{\left(\cdot \right)\left(\cdot \right)k}^{-(d,n)}+V\eta }\right) \nonumber \\ &&= \frac{\sum _{g=1}^{V}m(w_{d(\cdot )}^{g})N_{gdk}^{-(d,n)}+\alpha }{\sum _{g=1}^{V}m(w_{d(\cdot )}^{g})N_{gd(\cdot )}^{-(d,n)}+K\alpha }\cdot \frac{\sum _{d=1}^{D}m(w_{d(\cdot )}^{g})N_{gdk}^{-(d,n)}+\eta }{\sum _{g=1}^{V}\sum _{d=1}^{D}m(w_{d(\cdot )}^{g})N_{gdk}^{-(d,n)}+V\eta }. \nonumber\\
\end{eqnarray*}

Notice that, if all weights *m*( · ) = 1, this reduces to the standard LDA formulation in equation (3).

### gwSPADE weighting schemes

Let the frequency of a gene *w*^*g*^ in the count matrix be *p*(*w*^*g*^). The following are a few choices of weighting methods that can be applied.

- Inverse-frequency (IF) weighting: Down-weight genes that appear frequently across cell types, thereby emphasizing rare or selectively expressed genes. Biologically, this targets genes that may serve as unique markers. The IF weight is defined as:
  (7) \begin{eqnarray*}
m(w^{g})=\frac{1}{p(w^{g})}=\frac{\text{Counts of all genes}}{\text{Counts of gene }w^{g}}
\end{eqnarray*}
- Entropy weighting: Measures the uniformity of a gene’s expression across cell types. Genes with low entropy are more cell type specific and thus are assigned higher weights. This directly captures gene specificity. The entropy weight is calculated as:
  (8) \begin{eqnarray*}
m(w^{g})=-\log _{2}p(w^{g})=\log _{2}\frac{\text{Counts of all genes}}{\text{Counts of gene }w^{g}} \nonumber\\
\end{eqnarray*}
- Pointwise mutual information (PMI) weighting: Evaluates how much more (or less) a gene is associated with a particular cell type compared to what would be expected by chance. High PMI scores highlight genes with strong association to a particular cell type. The IF and entropy weighting metrics described above assign a constant value to each gene across all the spots. To relax this constraint, we can allow gene weights to vary across spots. PMI weighting has demonstrated effectiveness within the framework of Latent Semantic Indexing (LSI) [28], which is defined as
  (9) \begin{eqnarray*}
m(w_{d(\cdot )}^{g})&=& -\log _{2}\frac{p(w^{g}|d)}{p(w^{g})}\nonumber\\ &=& -\log _{2}\frac{\text{#[genes of type }g \text{ in spot } d\text{]}}{\text{#[genes of type }g]}
\end{eqnarray*}
- Term frequency-inverse document frequency (TF-IDF) weighting: A classic text mining measure, TF-IDF emphasizes genes that are frequent in a specific cell type but rare across others. It captures both local importance and global rarity, and has been used in scATAC-seq to mitigate sparsity by boosting signal from rare features. TF-IDF weighting combines the frequency of a gene in a given spot with the logarithmically scaled inverse of the number of spots in which that gene occurs across all spots, i.e.
  (10) \begin{eqnarray*}
&& m(w_{d(\cdot )}^{g})=\frac{\text{#[genes of type }g\text{ in spot }d\text{]}}{\text{#[genes in spot }d]}\nonumber\\ && \cdot \log _{2}\frac{\text{#[total spots]}}{\text{#[spots with gene of type }g]}.
\end{eqnarray*}
- Balanced Distributional Concentration weighting [23]: Inspired by supervised term weighting, BDC uses prior knowledge of cell type labels to weight genes with high discriminative power. It gives high weights to genes that are sharply expressed in one or a few cell types and low weights to ubiquitous or noisy genes. This aligns with the goal of highlighting cell type markers [24]. For a given *K*, we first fit a regular LDA model with the variational expectation-maximization (VEM) approach to estimate the gene expression probability matrix β as prior knowledge. This matrix is then used to compute the BDC weights as:
  (11) \begin{eqnarray*}
m\left(w^{g}\right)=1+(\sum _{k=1}^{K}\frac{\beta _{gk}}{\sum _{i=1}^{K}\beta _{gi}}\log \frac{\beta _{gk}}{\sum _{i=1}^{K}\beta _{gi}})/ \log K. \nonumber\\
\end{eqnarray*}
  This is a negative entropy measure: If gene *g* is uniformly expressed across all topics (nondiscriminative), entropy is high, so BDC is low. If gene *g* is specific to a few topics (discriminative), entropy is low, so BDC is high. Building on BDC, two additional weighting schemes can be extended:
- IF-BDC: It integrates IF weighting with BDC.
- Info-BDC: It integrates Entropy information weighting with BDC.

To ensure a fair comparison of these various weighting strategies, we employed a two-step normalization procedure. First, each weight was independently normalized using the max–min scale. If two weighting schemes were combined, the normalized weights from the first step were multiplied, and the resulting product was then normalized again using the max–min scale to produce the final weights.

The BDC weighting scheme enhances model performance by emphasizing genes that contribute most to topic separability. In topic modeling, analogous to feature selection in classification, it is beneficial to highlight dimensions (genes) that provide maximal information about the latent structure (cell types or topics). BDC evaluates the entropy of each gene in known cell types and favors genes with low entropy, those that are unevenly distributed and hence more specific to certain cell types. Statistically, this reduces the noise within the topic and sharpens the topic boundaries, leading to better convergence and more interpretable topics in LDA. Biologically, many housekeeping or structural genes are expressed across a broad range of cell types and contribute little to cell identity. BDC naturally down-weights these genes, allowing more specific markers, those indicative of functional or developmental specialization, to dominate topic inference. This mechanism mirrors strategies in other fields, such as TF-IDF in information retrieval [23, 24], where high-frequency but nondiscriminative terms are penalized. BDC, therefore, aligns with a broader principle of emphasizing context-specific features to improve deconvolution results.

### Evaluation metric

#### Gene selection

Regardless of reference-based or reference-free deconvolution, cell type marker genes always provide rich information for cell type deconvolution. Therefore, selecting genes that are more likely to be cell type-specific is essential in the deconvolution process. We first remove genes that are not detected in a sufficient number of spots, i.e. removing genes that occur in <1% of spots. We assume that the proportion of cell types will vary across spots, leading to their cell type-specific transcriptional profiles manifesting as overdispersed genes across spots in the dataset. We then select significantly overdispersed genes [29] or highly variable genes [30].

To evaluate the impact of the number of selected genes on deconvolution performance, we conducted an additional simulation using pseudo cell type expression profiles consisting of 10 000 genes across 8 cell types. High frequency genes were designed to be predominantly specific to particular cell types. We simulated 2000 spatial spots with random cell type compositions and an average of 10 000 counts per spot. We then performed deconvolution using the top 500, 1000, and 2000 most overdispersed genes and compared the results. The root mean squared error (RMSE) analysis shows that using only 500 genes leads to a wider range and greater variability in RMSE values. In contrast, selecting 1000 genes substantially reduces the RMSE range, yielding results very similar in range and median to those obtained using 2000 genes (Supplementary Fig. S9B). Additionally, the Pearson correlation coefficients (PCCs) between the deconvolved and true cell type transcriptomic profiles, as well as the estimated cell type proportions, demonstrate that 1000 genes achieve accuracy comparable to using 2000 genes (Supplementary Fig. S9A). Considering both accuracy and computational efficiency, we recommend using 1000 overdispersed genes, which typically represents ∼10% of the total number of genes in a ST dataset. This choice aligns with the default setting used in STdeconvolve [17]. Including too many genes can lead to poor topic separation and hinder model convergence; therefore, it is important to control the number of informative genes used for inference. In practice, we advise users to:

- Adjust the number of HVGs according to tissue heterogeneity and sequencing depth; for more complex tissues, a larger HVG set can help capture subtle sub-cell type variation.
- Optionally include known marker genes in addition to HVGs to ensure that biologically meaningful features are represented.
- Augment or customize the input gene set with additional genes of interest based on prior biological knowledge when needed.

#### Selecting the number of cell types

Following the idea of STdeconvolve [17], we do a grid search to determine the optimal number of cell type *K*. We start with *K* = 2 cell types, then increase *K* by one until a large number that depends on a specific tissue sample and also prior knowledge. For a given *K*, we calculate perplexity, a metric conventionally used in language models to measure model fit, which typically decreases as *K* increases.


(12) \begin{eqnarray*}
{\rm Perplexity}(D)=\exp \left\lbrace -\frac{\sum _{d=1}^{D}\log \left(p\left(w_{d}\right)\right)}{\sum _{d=1}^{D}N_{d}}\right\rbrace
\end{eqnarray*}

To avoid overfitting, we consider the number of rare cell types, defined as the number of cell types with an average proportion <5%. This default threshold of 5% was chosen because we found that the deconvolution accuracy of rare cell types with a mean proportion below 5% across spots is poor. However, this threshold can be adjusted to identify rare cell types when necessary. We aim to select the optimal number of cell types by balancing the two criteria.

The perplexity-based strategy in gwSPADE serves as a practical tool for selecting an appropriate number of topics *K* that balances model fit and interpretability. However, the final choice of *K* can and should also be guided by the biological context and specific research questions of interest. For example, users can leverage H&E histology images to assess tissue complexity before determining a suitable range for *K*. In the coronal section of the mouse brain dataset from 10× Visium, the H&E image clearly reveals multiple distinct cortical layers. Within each layer, the cell types are relatively homogeneous but may contain subtle sub-cell type heterogeneity, while different layers may contain entirely different major cell types. In such scenarios, researchers can use the known minimum number of anatomical layers as an initial estimate for *K* and increase it as needed to capture finer cell state distinctions. Therefore, perplexity-based selection provides a helpful quantitative starting point, but the final number of topics should reflect the researcher’s domain knowledge, tissue structure, and the biological resolution desired for a given study.

#### Annotation of deconvolved cell types

To establish the ground truth for cell type expression profiles in the simulated ST data derived from single cells, we compute the average expression of each gene across all cells belonging to a specific cell type. The deconvolved cell types are then matched with the ground truth by selecting the highest Pearson correlation between the cell type expression profiles.

When applying gwSPADE directly to a real ST dataset, identifying discriminative genes to interpret each cell type is crucial. Our approach involves two key steps:

- Gene selection and interpretation: After determining the optimal number of cell types, we scale the total gene expression counts within each deconvolved cell type to 1000. Genes exhibiting expression counts of 5 or higher are subsequently used to interpret the deconvolved cell types, ensuring that they are sufficiently significant to offer valuable insights into the cellular composition.
- Log2 fold-change analysis for sub cell type identification: To further distinguish deconvolved cell types and explore potential subtypes, we calculate the log2 fold-change for each gene in a specific deconvolved cell type compared to the average expression across other deconvolved cell types. Genes with a log2 fold-change >1 are considered as differentially expressed genes for the respective cell type. These differentially expressed genes highlight the unique transcriptional profile of each deconvolved cell type. For further analysis, we use these top differentially expressed genes in over-representation analysis (ORA) to assess whether known biological functions or processes are enriched in our gene list. This helps in understanding the functional relevance of the deconvolved cell types and their underlying biological processes [31, 32].

#### Comparison of different methods

To compare the performance between methods, we evaluate two aspects. For the deconvolved cell type expression profiles, we calculate both Pearson’s correlation and Spearman’s correlation for each ground truth cell type and the matched deconvolved cell type. Higher correlations indicate better matching to the ground truth cell types.

Additionally, when focusing on cell type compositions across spots, a high Pearson’s correlation for each matched cell type illustrates that these matched cell types have similar composition distributions across spots. The most common method to assess the accuracy of deconvolved composition within each spot is the RMSE. We compute the RMSE for each spot to assess accuracy, that is,


\begin{eqnarray*}
\text{RMSE}=\sqrt{\sum _{k=1}^{K}\left(\hat{\theta }_{k}-\theta _{k}\right)^{2}/K}
\end{eqnarray*}

where *K* is the total number of cell types, θ_*k*_ is the ground truth cell type proportion for cell type *k*, and $\hat{\theta }_{k}$ is the deconvolved cell type proportion matching the ground truth cell type *k*. To assess whether the distribution of RMSEs across all spots was significantly lower compared to other methods, we used a one-sided Diebold–Mariano test.

## Results

### Simulation

#### Model-based ST data simulation

**Figure 2.**
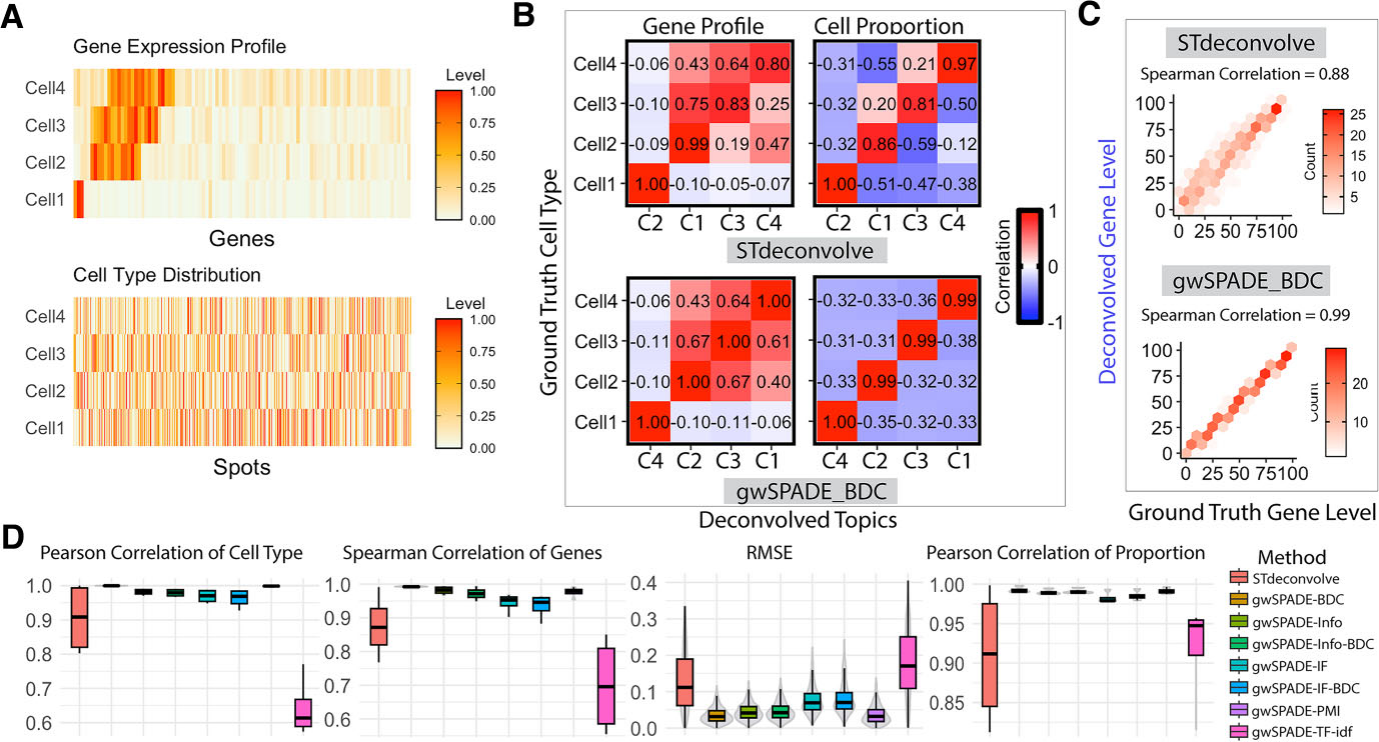
Deconvolution of Model-based ST data. (**A**) Ground truth gene expression profiles in each cell type (top) and ground truth cell type proportions across all spots (bottom). (**B**) PCC plots comparing the ground truth with deconvolved cell types. Top: STdeconvolve; Bottom: gwSPADE-BDC; Left: Transcriptional profiles of ground truth versus deconvolved cell types; Right: Cell type composition of ground truth versus deconvolved cell types. (**C**) Gene ranking comparison: the ranking of each gene based on its expression level in the deconvolved cell type transcriptional profiles, as compared to its rank in the matched ground-truth cell type transcriptional profiles, together with the corresponding SCC. Top: STdeconvolve; Bottom: gwSPADE-BDC. (**D**) (From left to right) Boxplot of PCCs between deconvolved cell types and the matched ground-truth gene expression profile. Boxplot of SCCs between deconvolved and the matched ground-truth gene expression profile. Boxplot of RMSEs of the deconvolved cell type proportions. Boxplot of PCCs between the proportions of deconvolved cell types and the matched ground-truth across all spots.

We first evaluated the performance of gwSPADE in recovering the proportional representations of cell types and their transcriptional profiles using model-based simulation data. In this model-based simulation, we did not rely on real gene expression data but instead generated synthetic data by sampling gene frequencies across cell types and cell type proportions across spots using Dirichlet distributions. This allowed us to construct a controlled environment with known ground truth for evaluating deconvolution performance. To simulate ST data, we generated pseudo cell type expression profiles for 100 genes, ensuring that high-frequency genes were predominantly associated with specific cell types. The distribution of cell types was simulated across 1000 spots with random assignments (Fig. 2A). This setup provided the ground truth for both transcriptional profiles and cell type proportions, with the count matrix sampled from a multinomial distribution [33], striking a balance between computational feasibility and interpretability. While this simplified setting does not fully replicate the complexity of real data, it is well-suited for evaluating the deconvolution performance of different methods. To assess how different weighting schemes improve deconvolution results, we applied gwSPADE with various weighting schemes (see “Materials and methods” section), as well as STdeconvolve, assuming *K* = 4 cell types in the simulated data. We then compared the deconvolved cell type expression profiles and cell type proportion distributions to the ground truth to measure the performance.

To infer the identities of deconvolved cell types for benchmarking, we matched their transcriptional profiles with the ground truth based on the highest Pearson correlation. We further evaluated the strength between the deconvolved cell type compositions and the ground truth across all cell types based on the best-performed weighting scheme, the BDC weighting scheme (Fig. 2B). Both the gene profile and cell type composition results show that gwSPADE-BDC outperformed STdeconvolve by showing higher correlations between the deconvolved results and the ground truth. Spearman correlation coefficient (SCC) between the deconvolved cell type’s transcriptional profile and the corresponding ground truth shows the superior performance of gwSPADE-BDC over STdeconvolve (Fig. 2C). Figure 2D shows the comparison results of STdeconvolve and gwSPADE with different weight schemes under different metrics: PCC, SCC, RMSE between the deconvolved and true cell type proportions across all spots, and PCC between the deconvolved and true cell type gene expression profile. Notably, the BDC weighting scheme shows the best performance across different metrics and much-improved performance compared with other metrics and with STdeconvolve. The results demonstrate the accuracy of gwSPADE in reconstructing both cell type-specific gene expression profiles and cell type compositions, with the BDC weighting scheme notably achieving a significantly lower RMSE compared with STdeconvolve and other weighting schemes (Diebold–Mariano *P*-value <2.2 × 10^−16^), reflecting improved precision.

#### Model-free ST data simulation

**Figure 3.**
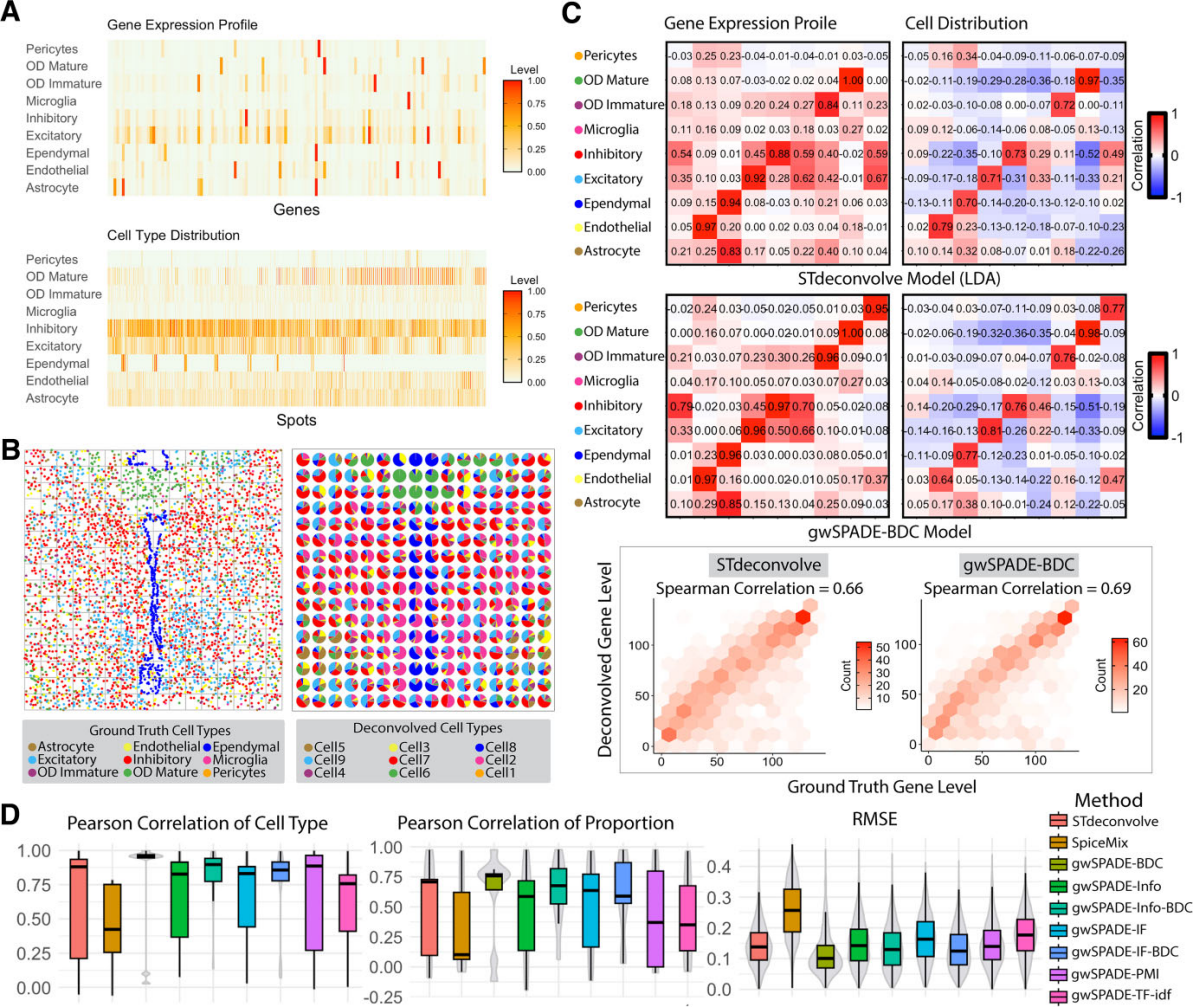
Deconvolution of MPOA simulated ST data. (**A**) (Top) Ground truth gene expression profile for each cell type in the MPOA; (bottom) ground truth cell type proportion for each spot in the MPOA. (**B**) (Left) Ground truth single-cell resolution MERFISH data from one section of the MPOA, partitioned into 100 μm^2^ grids (gray squares). (Right) Corresponding predicted cell type composition from gwSPADE-BDC. (**C**) (Top: STdeconvolve; Middle: gwSPADE-BDC) Heatmaps of Pearson’s correlations. The left panel shows transcriptional profiles comparing ground truth versus deconvolved cell types, while the right panel shows cell type composition comparing ground truth versus deconvolved cell types. (Bottom) Gene ranking based on expression levels in deconvolved cell type transcriptional profiles, compared to their rankings in the matched ground truth cell type transcriptional profiles, along with the corresponding SCC. (**D**) (From left to right) PCC between deconvolved cell types and matched ground truth transcriptional profiles, PCC between the proportions of deconvolved and ground truth cell types across spots, and RMSE of deconvolved cell type proportions per spot.

In this simulation, we simulated two ST datasets based on the single-cell datasets derived from the multiplex error-robust fluorescence *in situ* hybridization (MERFISH) data at the single-cell resolution [3] to evaluate the robustness and significance of gwSPADE-BDC. We first analyzed MERFISH data from the mouse medial preoptic area (MPOA) [34], where 135 selected genes were spatially mapped to distinguish major non-neuronal cell types and neuronal subtypes. The gene counts per cell were quantified to achieve single-cell resolution, enabling spatially resolved transcriptomic profiling. Subsequent clustering of gene expression data identified nine major cell types, including excitatory and inhibitory neurons. We selected one of the MPOA datasets containing the expression profiles of 49 138 cells from all 12 tissue sections of a female mouse. To simulate the ST data, we aggregated the single-cell gene expression into 100μm^2^ resolution nonoverlapping grids, resulting in 135 genes across 3072 grids. The proportion of cell types in each grid served as the ground truth for cell type distribution, while the average gene expression per cell type was used as the ground truth for cell type-specific gene profiles. Data visualization revealed the presence of high-frequency genes, suggesting that a weighted deconvolution may be beneficial (Fig. 3A).

We fixed the total number of cell types (*K* = 9) when performing STdeconvolve, SpiceMix, and gwSPADE with different weighting schemes. We identified the best-matched cell types for all deconvolved cell types by checking similarity through PCC between deconvolved and true cell type transcriptional profile. Compared to STdeconvolve, gwSPADE-BDC showed a higher PCC in both transcriptional profiles and cell type compositions across all spots (top two rows in Fig. 3C and Supplementary Fig. S1A), along with a stronger SCC in gene rankings (bottom row in Fig. 3C and Supplementary Fig. S1B). The deconvolved cell type compositions from gwSPADE-BDC closely resembled the real spatial patterns from the single cell resolution data (Fig. 3B). gwSPADE-BDC also outperformed other methods by showing a stronger PCC between the transcriptional profiles of each deconvolved cell type and the matched ground truth cell type across genes per cell type (Fig. 3D, left), along with higher PCCs in cell type compositions across simulated grids (Fig. 3D, middle). gwSPADE-BDC also shows the smallest RMSE of the deconvolved cell type proportions compared to the ground truth across simulated grids (Fig. 3D), showing significantly better performance compared to other weighting schemes (Diebold–Mariano *P*-value <2.2 × 10^−16^).

**Figure 4.**
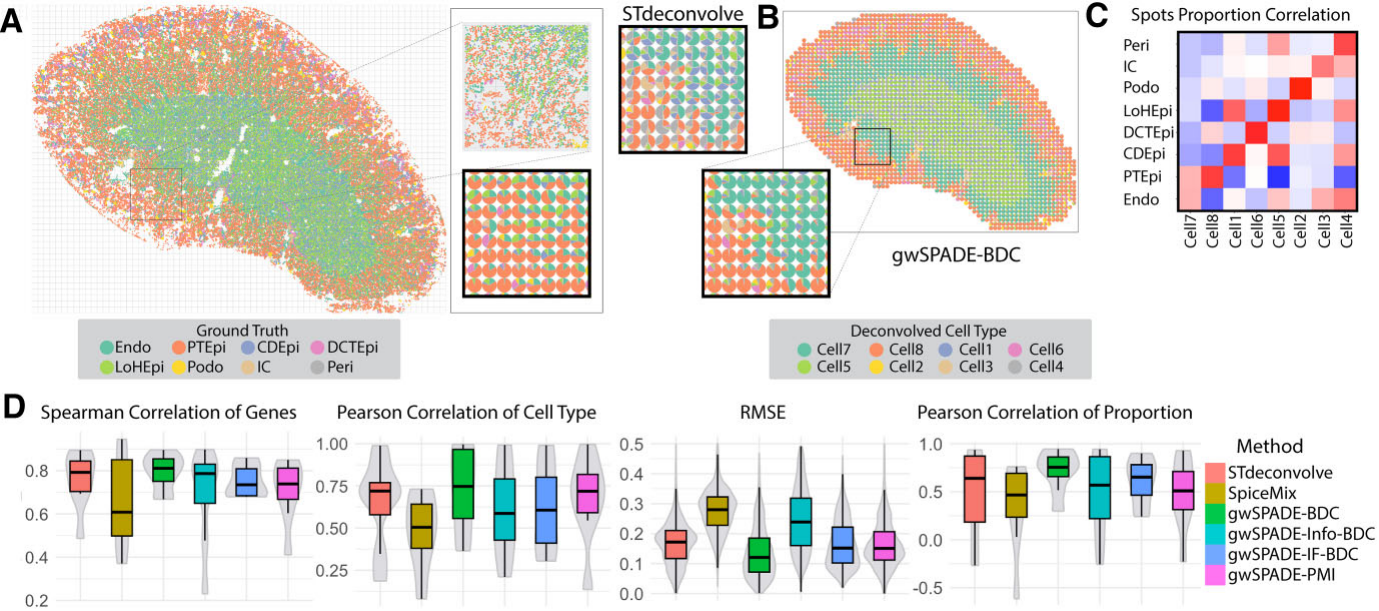
Deconvolution of the simulated ST data derived from the MK single-cell data. (**A**) The spatial image of the single-cell resolution MK MERFISH data (left), a zoomed-in view of a selected area (top-right), and the pie chart of the cell type proportion after merging single cells into spots (bottom, right). (**B**) The deconvolved cell type proportion pie chart by STdeconvolve for the selected area (top, left); the deconvolution view of gwSPADE-BDC (right), including a zoomed-in view of the same selected area (bottom, left). (**C**) Heatmap showing the Pearson correlations between ground truth cell types and their matched deconvolved cell types. (**D**) Boxplots (from left to right) of SCC and PCC between deconvolved cell types and their matched ground truth transcriptional profiles, RMSE of deconvolved cell type proportions across all spots, and PCC between the proportions of deconvolved cell types and matched ground truth cell types across all spots. Abbreviations: endothelial cell (Endo), epithelial cell of the proximal tubule (PTEpi), immune cell (IC), collecting duct epithelial cell(CDEpi), distal convoluted tubule epithelial cell (DCTEpi), loop of Henle epithelial cell (LoHEpi), pericyte (Peri), and podocyte (Podo).

We then examined another publicly available MERFISH single-cell dataset from mouse kidney (MK) [35], which contains the expression of 307 genes across 126 547 cells, annotated into eight distinct cell types. Using a similar approach, we simulated ST data by dividing the single-cell MK data into 2472 spatially contiguous grids and aggregated the gene expression of cells within each grid to form the spot-level data (Fig. 4A). This process yielded the ground truth for both cell type distribution and cell type transcriptional profiles based on the average gene expression for each cell type.

Next, we applied the gwSPADE model with various weighting schemes, as well as STdeconvolve, to the simulated MK ST data, aiming to infer the cell type compositions for each spot, fixing the number of cell types as eight. Using the PCC of cell type gene expression profiles, we matched the deconvolved cell types for benchmarking (Supplementary Fig. S2A). While correlation analyses indicated how closely the deconvolved cell type compositions matched the ground truth (Fig. 4C), pie chart visualizations of the deconvolved cell type distributions revealed that gwSPADE-BDC exhibited proportion patterns more similar to the ground truth than STdeconvolve (Fig. 4B and Supplementary Fig. S2B). As expected, gwSPADE-BDC demonstrated stronger correlations for each matched deconvolved cell type against the ground truth gene expression profiles as well as between the deconvolved and ground truth cell type proportions across all spots (Fig. 4D and Supplementary Fig. S2C). Additionally, it achieved a significantly lower RMSE for cell type proportions per grid compared to STdeconvolve (Diebold–Mariano *P-*value <2.2 × 10^−16^) and SpiceMix (Diebold–Mariano *P*-value <2.2 × 10^−16^). Beyond its overall outstanding performance, gwSPADE-BDC consistently demonstrated superior performance with high PCC for both gene expression profiles and cell type proportions across all spots (Fig. 4D). In summary, in all simulated ST datasets, gwSPADE with the BDC weighting scheme significantly outperformed other weighting schemes as well as STdeconvolve and SpiceMix. Therefore, we recommend using the BDC weighting scheme when applying gwSPADE.

### Benchmark with Real ST data deconvolution

In addition to demonstrating superior performance in simulated ST data, gwSPADE has proven to be highly compatible with a variety of ST platforms in different real data analyses. Following simulation results, we applied gwSPADE-BDC to four published ST datasets: two from the ST platform, one from 10× Visium, and one from DBiT-seq.

#### Mouse olfactory bulb ST deconvolution

**Figure 5.**
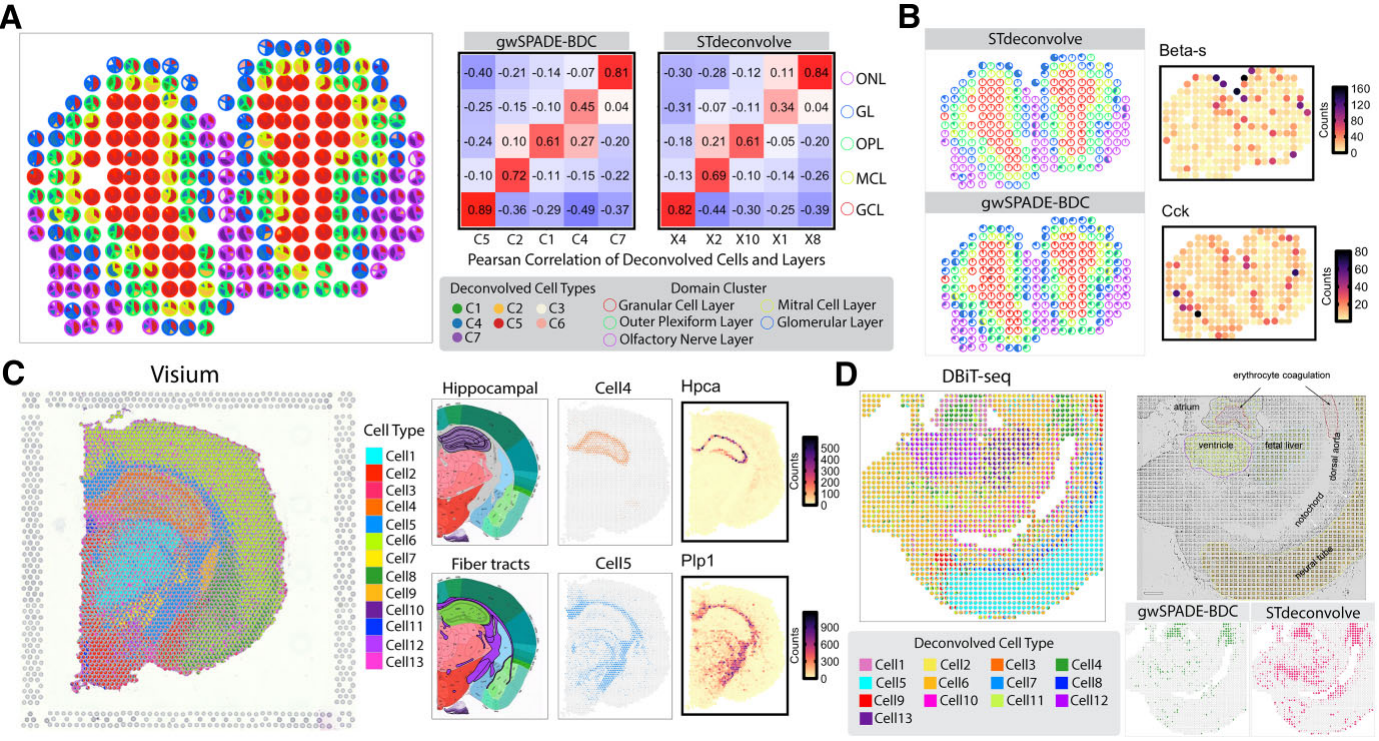
Deconvolution of MOB and mouse brain ST data. (**A**) Left: deconvolved cell type proportions for the MOB data, represented as pie charts for each spot. Spots are outlined with colors based on cluster assignments corresponding to MOB layers. Right: Heatmap showing the Pearson’s correlations between the layer assignments and the deconvolved cell type proportions across spots, with the diagonal indicating the best matches. (**B**) Highlights of the identified deconvolved cell type in the GL and corresponding top frequency gene from each method. (top: STdeconvolve; bottom: gwSPADE-BDC). Pie charts (left) represent the distribution of the matched deconvolved cell type proportions in GL, using consistent colors with spots outlined in the same colors as in panel (A). The two figures on the right display the gene count distributions for the top-frequency gene associated with the GL-matched deconvolved cell type identified by each method. (**C**) Deconvolved cell type proportions for the mouse brain data from 10× Visium, represented as pie charts for each spot (left one). The six figures in the right, from left to right: histological annotation regions, highlighted deconvolved cell types corresponding to the annotation regions, and the corresponding top differentially expressed gene counts across all spots (top: hippocampus; Bottom: fiber tracts). (**D**) Deconvolved cell type proportions for DBiT-seq data of the lower body of an E11 mouse embryo (left); and tissue types identified from the original paper’s clustering (top right) and the highlight of the distribution of the deconvolved cell types associated with erythrocyte coagulation, comparing gwSPADE-BDC and STdeconvolve results (bottom right).

Our initial analysis focused on the mouse main olfactory bulb (MOB) dataset [1]. This dataset includes five primary symmetric layers [36], identified through general histology: the granule cell layer (GCL), the mitral cell layer (MCL), the outer plexiform layer (OPL), the glomerular layer (GL), and the nerve layer (ONL) (Supplementary Fig. S3A). In gwSPADE-BDC, we determined that the optimal number of cell types was *K* = 7 (Supplementary Fig. S3B). To evaluate the accuracy of deconvolved cell types between gwSPADE-BDC and STdeconvolve, we measured the correlation between the deconvolved cell type proportions and the spots’ layer assignments. This allowed us to align the deconvolved cell types to the respective layers and check if the identified cell types match the expected layer distribution. The cell type compositions predicted by gwSPADE-BDC accurately reflected the layered structure of the MOB. From the correlation plot (Fig. 5A), it is evident that gwSPADE-BDC significantly improved the accuracy of deconvolved cell types, particularly for the GL layer. Specifically, the deconvolved cell type C4 from gwSPADE-BDC, enriched in GL, showed a clear and consistent pattern with the GL structure (correlation = 0.45). In contrast, the matched deconvolved cell type X1 from STdeconvolve was only found in a few spots matched with GL (correlation = 0.34), failing to establish a clear pattern consistent with the GL layer (Fig. 5B). Furthermore, when examining the high frequency genes within each corresponding deconvolved cell type, the top expressed gene *Beta-s* in topic X1 by STdeconvolve showed poor alignment with the GL layer. In contrast, the top gene *Cck* in topic C4 by gwSPADE-BDC demonstrated a strong match with the GL layer. Note that gene *Cck* is also a marker gene for GL, further showing the practical relevance of the proposed deconvolution method [37].

#### Mouse brain ST deconvolution

We next applied gwSPADE-BDC to the ST data from a coronal section of the mouse brain using 10× Visium [38]. In 10× Visium, mRNAs from tissue sections are captured on an array of DNA-barcoded spots, each with a resolution of 55 μm^2^. Primers with spatial barcodes enable RNA-sequencing measurements with precise spatial information. Using gwSPADE-BDC, we identified the optimal number of deconvolved cell types as *K* = 13 (Supplementary Fig. S4A), and the results revealed spatially distinct patterns that aligned with known brain structures from the Allen Brain Atlas [39], such as fiber tracts and hippocampal regions. Analysis of top genes further supported these findings by identifying top differential expressed genes for the deconvolved cell types. For instance, we recognized *Hpcs*, a strong marker for the hippocampus [40], and *Plp1*, a well-established marker for oligodendrocytes [41]—the cells responsible for myelination in the central nervous system, including fiber tracts (Supplementary Fig. S4B). These top differentially expressed genes showed clear and discernible spatial patterns consistent with known anatomical features.

We then applied gwSPADE-BDC to the lower body of the E11 mouse embryo ST data from DBiT-seq [42], which has a 25 μm^2^ resolution. DBiT-seq uses a microfluidics system to deliver DNA barcodes in a precisely controlled pattern, applying two sets of DNA barcodes perpendicularly to create a grid of unique barcodes. gwSPADE-BDC identified an optimal number of cell types, *K* = 13 (Supplementary Fig. S5A), which matches the number of transcriptionally and spatially distinct features described by the original authors [42], including the atrium, ventricle, liver, and blood vessels containing erythrocyte coagulation (Fig. 5D). The distribution of deconvolved cell types from gwSPADE-BDC aligned well with these distinct spatial features, consistent with the histological annotations in the original findings. For example, we observed clear spatial patterns for the ventricle, fetal liver, and neural tube (Supplementary Fig. S5B). Additionally, the top differentially expressed genes in each deconvolved cell type included expected markers for the corresponding anatomical features (Supplementary Fig. S5C). While STdeconvolve identified deconvolved cell types with relatively consistent patterns, gwSPADE-BDC demonstrated clearer improvements, particularly by reducing misassignments and preventing an overrepresentation of cell types in unrelated or obstructive regions, especially in areas associated with erythrocyte coagulation (Fig. 5D).

#### Human PDAC ST data deconvolution

For the human pancreatic ductal adenocarcinoma (PDAC) ST data from microarray slides [43], we evaluated the competitive performance of gwSPADE-BDC against deconvolution methods that require marker gene lists, such as CARDfree [11] and SMART [44]. Though CARDfree is described as reference-free, it still requires a marker gene list as input. SMART, a semi-reference-based method, employs a keyword-assisted LDA model for deconvolution and also relies on a marker gene list. In contrast to SMART, which directly assigns deconvolved cell types based on the provided markers, CARDfree is an unsupervised model that incorporates marker genes as selected features in the count matrix, leveraging a conditional autoregressive-based non-negative matrix factorization model. However, CARDfree can only identify as many cell types as specified in the marker list and does not directly assign them to known cell types. For consistency, we used the same marker genes for both methods.

**Figure 6.**
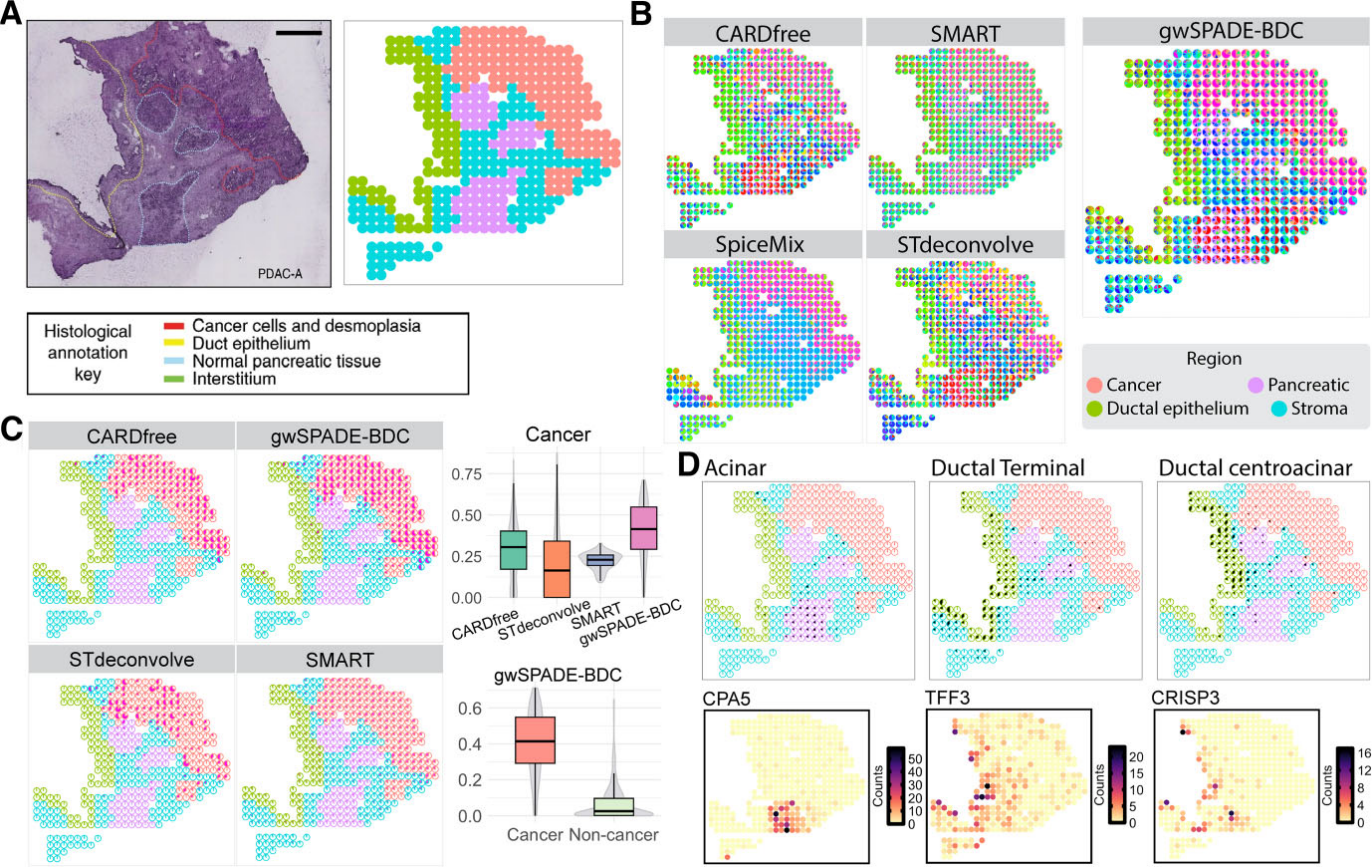
Deconvolution of the PDAC ST data. (**A**) (Left) Histology staining image of the tissue. (Right) Regions annotated from the original study. (**B**) Pie charts showing inferred cell type compositions in each spot using different deconvolution methods, including CARDfree, SMART, SpiceMix, STdeconvolve, and gwSPADE-BDC. Spots are outlined with colors based on annotations. The patterns of gwSPADE-BDC demonstrate a closer match to the ground truth in panel A (right). (**C**) (Left) Highlights of the identified deconvolved cell types for tumors from CARDfree, SMART, STdeconvolve, and gwSPADE-BDC, represented in pie charts. (Right) Top: Boxplot of the proportions of identified deconvolved tumor cell types in cancer-annotated regions. Bottom: Comparison of cell type proportions inferred by gwSPADE-BDC in cancer regions versus noncancer regions. (**D**) Top: Proportion of each cell type inferred by gwSPADE-BDC displayed at each spatial location. Bottom: Expression pattern of the corresponding identified cell type-specific top differential expressed genes.

The PDAC data consists of multiple tissue regions, cancer, ductal, pancreatic, and stroma, annotated by histologists based on H&E staining (Fig. 6A). We applied four deconvolution methods to this data, focusing on 1379 genes from CARDfree marker gene list of the PDAC dataset [11]. Since CARDfree predicts a number of cell types equal to the number in the marker gene list and relies solely on the expression counts of these marker genes, we ensured a fair comparison by using the same marker gene-specific count matrix as input for SMART, STdeconvolve, and gwSPADE-BDC, setting the number of cell types to 20 to match the marker gene list.

As anticipated, gwSPADE-BDC performed the best in identifying major cell types within their corresponding regions. In the cancerous region, gwSPADE-BDC provided the clearest spatial patterns and high proportions of deconvolved cancer clone cells, outperforming the other methods. While SpiceMix identified the highest proportions of deconvolved cancer clone cells, likely due to the spatial continuity of the cancer region enhancing the performance of its spatial network, it also mistakenly assigned higher cancer cell proportions to noncancerous areas (Supplementary Fig. S6A). Specifically, gwSPADE-BDC predicted significantly higher proportions of cancer cells in the cancerous region compared to noncancerous areas (*t*-test *P*-value = 2.2 × 10^−16^) (Fig. 6C). Additionally, gwSPADE-BDC accurately identified cell types in various regions through top gene analysis. In the ductal region, we successfully identified both ductal terminal and centroacinar cells based on the top differentially expressed genes of the deconvolved cell types, *TFF3*, a marker gene for ductal terminal cells, and *CRISP3*, a marker gene for ductal centroacinar cells. In the pancreatic region, we identified acinar cells using their top deconvolved gene, *PNLIPRP1* (Fig. 6D). Furthermore, gwSPADE-BDC identified acinar-adjacent cell types, which shared similar marker genes but diverged in functional pathways, as revealed by gene enrichment analysis (Supplementary Fig. S6C and D). Otherwise, SpiceMix performed poorly in regions lacking spatial continuity, where it failed to effectively distinguish distinct cell type patterns (Supplementary Fig. S6B).

When comparing gwSPADE-BDC with STdeconvolve for detailed deconvolution, notable differences in gene weighting emerge. In STdeconvolve, certain top-frequency genes tend to dominate the deconvolved cell types without providing clear discriminatory power. For example, the high-frequency gene *S100A6* appeared among the top 10 most frequent genes in 13 deconvolved cell types in STdeconvolve, thereby overshadowing other cell type-specific markers. In contrast, gwSPADE-BDC effectively downweighted *S100A6*, reducing its dominance to only five deconvolved cell types (see Supplementary Fig. S7B). Additionally, gwSPADE-BDC upweighted discriminative genes that might not be highly expressed overall. Whereas STdeconvolve’s top 10 frequent genes were generally high-expression genes across all spots, gwSPADE-BDC balanced this by upweighting lower-expression, yet informative genes, and downweighting overly dominant ones (see Supplementary Fig. S7A). This refined weighting strategy prevented high-expression genes from skewing the deconvolution process, resulting in more distinct and accurate deconvolved cell types. These findings underscore the critical role of gene weighting in achieving improved deconvolution performance.

#### Deconvolution of cell type-specific spots to further identify sub-cell types

**Figure 7.**
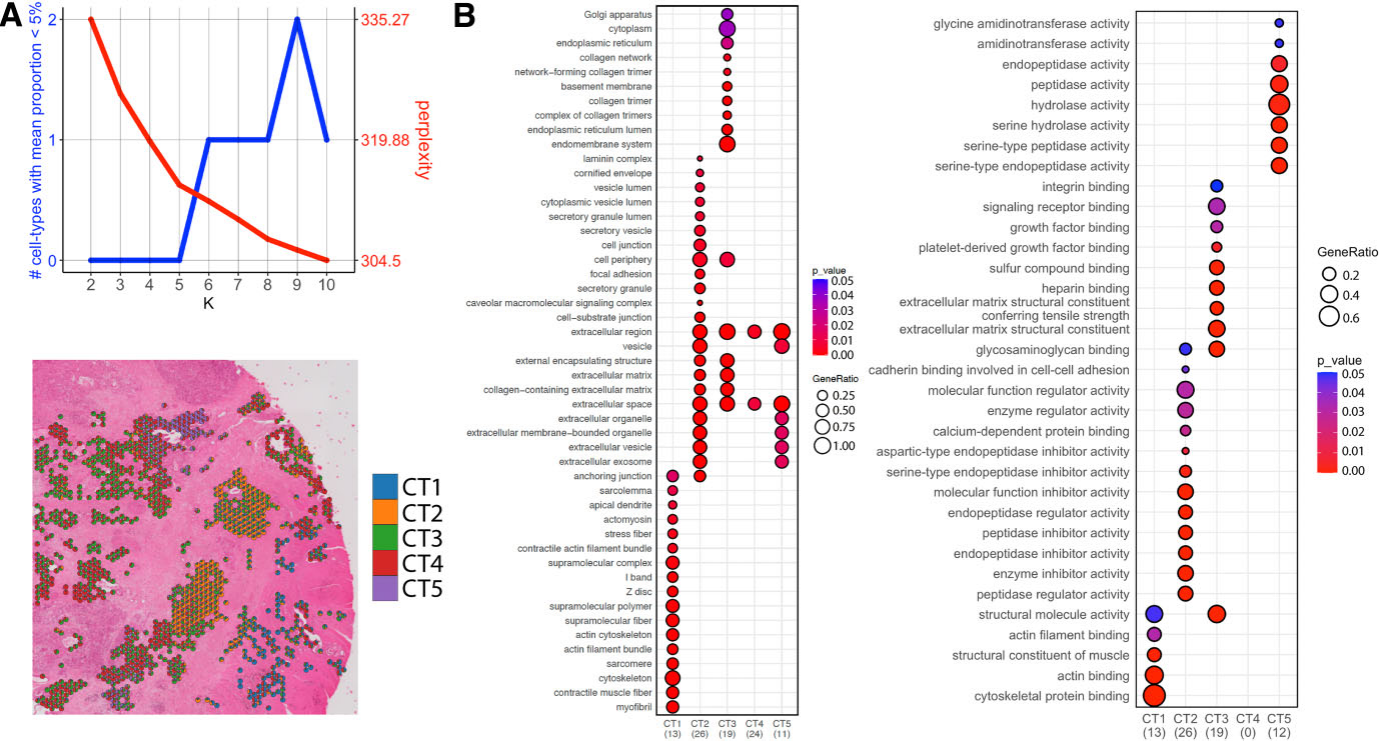
Cell subtype deconvolution of the PDAC ductal purity spots (>60%). (**A**) (Top) Plot of the perplexity score and the number of cell-types with mean proportion <5% shows the optimal number of cell type as five. (Bottom) Histology staining image of the tissue with pie charts showing inferred sub-cell-type compositions in spots with the ductal cell proportion >60%. (**B**) Pathway enrichment analysis of unique top expressed genes from inferred sub-cell types based on the GO terms. The number below each sub-cell type (denoted as CT) represents the number of unique genes in that sub-cell type overlapped with genes in that GO term pathway. The GeneRatio represents the proportion of genes in a specific GO term relative to the overlapped genes in that sub-cell type (the number below CT). (Left) GO term CC ontology. (Right) GO term MF ontology.

One of the advantages of a reference-free deconvolution method is that it can identify cell types without single cell reference information. In our previous deconvolution analysis for a PDAC data derived from a PDAC patient who underwent surgical resection (without neoadjuvant chemotherapy) [45], we used a reference-based deconvolution method called Cottrazm [46] to identify major cell types. We named this PDAC dataset as nontreated PDAC (NT-PDAC) ST data. The reference dataset was obtained from our prior study [47]. Ductal cells largely represent the tumor cell compartment in PDAC. As previous studies have identified unique transcriptional subtypes within the tumor compartment of PDAC [48], we then focused on spots with the deconvolved ductal cell proportion >60% to identify potential sub-cell types without any further reference information about the potential sub-cell types. To achieve this, we implemented the gwSPADE-BDC algorithm, which revealed five distinct sub-cell types within the tumor compartment revealed by the perplexity plot (Fig. 7A). For each sub-cell type, we identified a set of unique top expressed genes and conducted GO term enrichment analysis across the Cellular Component (CC), Molecular Function (MF), and Biological Process (BP) ontologies. The GO–CC and GO–MF results are shown in Fig. 7B, while the GO–BP result is shown in Supplementary Fig. S8 due to its large size. The results unveil distinct roles in cytoskeletal dynamics, extracellular matrix (ECM) remodeling, angiogenesis, ion transport, and digestion, offering novel insights into PDAC cell population’s unique spatial relationships with the tumor microenvironment.

The five sub-cell types derived from spots with >60% ductal purity in the PDAC sample have meaningful biological implications. Sub-cell type 1 is enriched in CC terms like “myofibril” and “actin cytoskeleton,” MF terms including “actin binding,” and BP terms such as “muscle contraction” and “cytoskeleton organization.” This suggests a myofibroblast-like role, leveraging actin-based contractility to maintain structural integrity and drive mechanical forces [49]. In PDAC, this enhances tumor cell migration and metastasis [50]. Targeting actin polymerization (e.g. cytochalasin D) could disrupt motility, while the muscle-like phenotype hints at a novel stromal contributor to TME stiffness [51]. On review of the spatial location of these cells on the histology, this sub-cell type 1 was notably enriched in the tumor cells invading into the wall of the smooth muscle, suggesting a more invasive property of this tumor subtype.

Sub-cell type 2 features CC terms like “extracellular exosome” and “focal adhesion,” MF terms such as “cadherin binding” and “peptidase inhibitor activity,” and BP terms including “cell migration” and “plasminogen activation.” This profile indicates a stromal or cancer cell modulating adhesion and ECM dynamics via exosomes and protease inhibition (e.g. SERPINE1) [52, 53]. Its dual role in physical (migration) and biochemical (ECM remodeling) processes suggests a key player in invasion. FAK inhibitors or exosome blockers (e.g. GW4869) could target this sub-cell type, revealing a novel synergy between vesicle signaling and proteolysis in PDAC. Interestingly, sub-cell type 2 was enriched in histological regions that correlated with poorly differentiated cell character, which may be indicative of an epithelial-to-mesenchymal phenotype that has been commonly described in PDAC.

Sub-cell type 3 shows CC terms like “collagen trimer” and “basement membrane,” MF terms including “extracellular matrix structural constituent” and “integrin binding,” and BP terms such as “extracellular matrix organization” and “blood vessel development.” This points to a fibroblast-like role in ECM remodeling and angiogenesis, critical for tumor growth and metastasis [54, 55]. Integrin or anti-angiogenic therapies (e.g. bevacizumab) could destabilize the TME. The novel integration of ECM stiffening with vascularization underscores a dual support mechanism in PDAC desmoplasia [51].

Sub-cell type 4 is enriched in CC terms “extracellular region” and BP terms “monoatomic ion transmembrane transport” and “positive regulation of sodium ion transmembrane transporter activity” (no enrichment found in GO-MF). This suggests a role in sodium ion homeostasis (e.g. via Na^+^/K^+^-ATPase), potentially dysregulated in cancer to support proliferation and migration [56]. Sodium channel blockers (e.g. amiloride) could impair tumor dynamics. The extracellular focus hints at secreted ion regulators [57].

Sub-cell type 5 integrates CC terms like “extracellular vesicle,” MF terms such as “serine-type endopeptidase activity,” and BP terms including “digestion” and “regulation of nitric oxide mediated signal transduction.” This reflects an acinar-like role in protease secretion (e.g. trypsin) and NO signaling (e.g. NOS2), aiding ECM degradation and vascular/immune modulation in PDAC [58, 59]. Protease or iNOS inhibitors could limit invasion and immune evasion. The novel coupling of exocrine function with NO-mediated TME effects suggests a unique progression driver [60]. We noted that sub-cell type 5 correlated with a more well-differentiated histology, suggesting an association of these functions with tumor cells that more closely mimic a ductal structure.

These sub-cell types collectively shape PDAC’s aggressive TME: sub-cell type 1 drives motility, sub-cell type 2 regulates ECM/adhesion, sub-cell type 3 supports vascularization, sub-cell type 4 modulates homeostasis, and sub-cell type 5 links digestion to signaling. Their coherent roles across GO ontologies highlight PDAC heterogeneity, with novel insights like exosome-proteolysis synergy (sub-cell type 2) and digestion-NO interplay (sub-cell type 5). Targeting these processes offers promising strategies to disrupt PDAC progression. Future studies will investigate if these terms represent a close association of these tumor cells with myofibroblasts or other subtypes of cancer associated fibroblasts as well as other key TME components that we and others have previously described as important for driving aggressive phenotypes in PDAC [47, 61, 62].

## Discussion

Exploring the precise composition of cell types at spot-level resolution in ST data is essential for advancing our understanding of cell type-specific spatial patterns and transcriptional landscapes. The reference-free deconvolution method we developed provides a robust approach for uncovering transcriptional heterogeneity in ST data without relying on pre-defined reference profiles. Unlike traditional deconvolution methods that require well-annotated marker genes or known cell type profiles (e.g. CARD [11]), our method employs data-driven statistical modeling to infer cell-type-specific transcriptional signatures directly from the data. This enhances its applicability, particularly in cases where reference profiles are unavailable or incomplete, such as novel tissue types or rare pathological conditions. Additionally, it can be used to deconvolve a specific cell type (following reference-based deconvolution) with high purity across spatial locations, enabling the discovery of subtle, previously unrecognized heterogeneity within that cell type, as demonstrated in the real data analysis.

In simulated ST datasets, whether derived from model-based data or ideal single-cell data like MERFISH, gwSPADE consistently outperformed STdeconvolve SpiceMix by identifying cell types with spatially coherent gene expression patterns, achieving lower mean squared errors and higher correlations. For instance, while STdeconvolve often prioritizes genes with universally high expression across spots, our method effectively extracts subtle, low-expression genes that are locally enriched, as observed in the transcriptional profiles of ductal and acinar cell types. This improved resolution enhances our understanding of spatial tissue organization and cell-cell interactions, which are crucial for studying biological processes such as tissue development and cancer progression.

In our benchmarking experiments, we focused on using a fixed *K* equal to the number of major cell types present in each dataset to allow direct performance comparisons. However, we note that real tissues often contain additional layers of sub-cell-type heterogeneity, such as excitatory, inhibitory, or astrocyte subpopulations, which our model is capable of resolving. To guide users in practical applications, gwSPADE provides a perplexity-based strategy to select an appropriate *K* that balances model fit and interpretability. Importantly, this serves as a starting point: the final choice of *K* can and should be informed by biological knowledge, histological context, and specific research goals.

In real ST data analyses, gwSPADE provided better alignment with regions annotated from H&E staining in both mOB and mouse embryo samples across various platforms and resolutions. For the PDAC dataset, gwSPADE outperformed other deconvolution methods that rely on marker gene lists, such as SMART and CARDfree. SMART, being semi-supervised, heavily depends on cell type-specific marker genes to identify known cell types, while CARDfree uses marker genes to estimate the number of deconvolved cell types but fails to directly match them, as it operates on a non-negative matrix factorization model. This often results in inaccurate transcriptional profiles. Both methods struggle with incomplete marker gene lists, leading to mixed or missing cell types. While SpiceMix was able to identify a high proportion of cancer cells, it performed poorly in regions that lacked sufficient spatial continuity, limiting its ability to accurately resolve cell types. Furthermore, when the number of cell types exceeds expectations, the resulting deconvolved expression profiles become overly sparse [16], making interpretation of the results challenging. In contrast, gwSPADE is a fully unsupervised generative probabilistic model that requires no additional input, enabling a more robust deconvoluton result. Furthermore, gwSPADE overcomes a key limitation of STdeconvolve, a standard LDA-based model, where high-frequency genes arbitrarily dominate multiple cell types, biasing the deconvolution results. Our findings demonstrate that gwSPADE provides enhanced cell type compositions and transcriptional profiles across various ST platforms using both simulated and real datasets.

Despite its advantages, gwSPADE, like the LDA model in STdeconvolve, has limitations. Its performance depends on dataset size, particularly the number of spots and genes [63], and the selection of genes affects deconvolution results. Future improvements may involve optimizing computational efficiency through adopting scalable algorithms. Additionally, like STdeconvolve, gwSPADE does not integrate spatial information and assumes that spots are independent. Further advancements that incorporate spatial information into the deconvolution process warrant investigation.

Overall, gwSPADE provides a robust reference-free deconvolution method for ST data, offering an alternative to approaches relying on scRNA-seq reference profiles. By incorporating an effective weighting scheme, gwSPADE-BDC achieves improved accuracy and interpretability compared to other reference-free deconvolution methods and performs on par with some of the reference-based approaches. It holds great promise for unsupervised clustering, subtype identification, and data visualization, offering a valuable tool for investigating tissue heterogeneity and gene expression variation across diverse ST platforms. By enabling improved cell type discovery, gwSPADE broadens the utility of ST technologies in fields such as developmental biology, disease modeling, and therapeutic research.

## Supplementary Material

gkaf966_Supplemental_File

## Data Availability

The MERFISH dataset of the mouse medial preoptic area [34] is available for download from https://datadryad.org/stash/dataset/doi:10.5061/dryad.8t8s248/. The MK dataset [35] are available for download from https://figshare.com/projects/MERFISH_mouse_comparison_study/134213. Data and H&E images for all MOB replicates [1] are available for download at https://www.spatialresearch.org/resources-published-datasets/doi-10-1126science-aaf2403/. The 10× Visium coronal section of the mouse brain dataset [38] is available for download at https://www.10xgenomics.com/resources/datasets/mouse-brain-section-coronal-1-standard-1-1-0. DBiT-seq dataset of E11 mouse embryo lower body (GSM4364242_E11-1L) [42] is available for download at https://www.ncbi.nlm.nih.gov/geo/query/acc.cgi?acc=GSE137986. The PDAC data were obtained from sample A of the PDAC dataset and are available at the Gene Expression Omnibus (accession number GSE111672) [43]. The NT-PDAC human pancreatic cancer data [47] can be found through dbGaP with accession number PRJNA1124001 and the image files are available at https://zenodo.org/records/13379726.

## Code availability

Codes of gwSPADE are available in the GitHub repositories at https://github.com/Cui-STT-Lab/gwSPADE and Zenodo at https://zenodo.org/records/16953533.
