# Supplementary material for: gwSPADE: gene frequency-weighted reference-free deconvolution in spatial transcriptomics
Source: Nucleic Acids Res. 2025 Sep 26;53(18):gkaf966. doi: 10.1093/nar/gkaf966 (PMC12464827; doi:10.1093/nar/gkaf966)
Supplement: gkaf966_Supplemental_File [file gkaf966_supplemental_file.pdf]

Supplementary material for "gwSPADE: Gene Frequency-weighted Reference-free Deconvolution in Spatial Transcriptomics"

Aoqi Xie[1], Nina G. Steel[2,3,4,5], and Yuehua Cui*[1]

[1]*Department of Statistics and Probability, Michigan State University, East Lansing, MI.*
[2]*Department of Surgery, Henry Ford Michigan State University Pancreatic Cancer Center, 4-Henry Ford Health, Detroit MI*
[3]*Department of Pharmacology and Toxicology, Michigan State University, East Lansing MI*
[4]*Department of Oncology, Wayne State University, Detroit, MI.*
[5]*Division of Gastroenterology and Hepatology, Department of Internal Medicine, College of Medicine, University of Cincinnati, Cincinnati OH*

1 Estimation with the collapsed Gibbs sampler

Latent Dirichlet Allocation (LDA) has been proposed to identify latent topics for a given set of documents[1]. In the specific context of spatial transcriptomics, this model can be analogously applied[2]. The posterior distribution of latent variables based on the observed gene expression data can be obtained as,

$$p(\theta, z|w, \alpha, \beta) = \frac{p(\theta, z, w|\alpha, \beta)}{p(w|\alpha, \beta)} = \prod_{d=1}^{D} p(\theta_d, z|w, \alpha, \beta). \tag{1}$$

where w is the collection of the corresponding genes in the gene expression matrix, z is the collection of assigned cell types of corresponding genes, θ is the distribution of cell types in all spots, θ_d is the distribution of cell types in spot d, α is the uniform Dirichlet scale parameter for θ, β is the matrix of relative gene expressions for all cell types. β and θ can be estimated with two popular methods: the Variational EM algorithm and the collapsed Gibbs sampler. In our gene frequency-weighted LDA model, we implemented the latter one. Next, we briefly introduce the algorithms.

We assume that the probability of each gene in cell type k, denoted as β_k, follows a uniform Dirichlet distribution with scaling parameter η, i.e. $\beta_k \sim \text{Dir}(\eta)$. Then, the posterior distribution can be obtained as:

$$p(\theta, z, \beta|w, \alpha, \eta) = \frac{p(\theta, z, w, \beta|\alpha, \eta)}{p(w|\alpha, \eta)} \tag{2}$$

Next, we first show the basic Gibbs sampler for LDA model by calculating the complete conditional distributions as follows:

*Corresponding author. E-mail: cuiy@msu.edu;

- The conditional distribution of cell type (component) proportions θ_d is given as

$$\begin{aligned}
p(\theta_d|z, \theta_{-d}, w, \beta) &= p(\theta_d|z_d, \alpha) \propto \prod_{n=1}^{N_d} p(z_{d,n}|\theta_d)p(\theta_d|\alpha) \\
&\propto \prod_{n=1}^{N_d}\prod_{k=1}^{K} \theta_{d,k}^{z_{d,n}^k} \prod_{k=1}^{K} \theta_{d,k}^{\alpha-1} \\
&= \prod_{k=1}^{K} \theta_{d,k}^{\alpha-1+\sum_{n=1}^{N_d} z_{d,n}^k} \\
&= \mathrm{Dir}(\alpha + \sum_{n=1}^{N_d} z_{d,n}) \\
&= \mathrm{Dir}(\alpha + z_d)
\end{aligned}$$

- The conditional distribution of gene expression β_k is given as

$$\begin{aligned}
p(\beta_k|z, \theta, w, \beta_{-k}) &= p(\beta_k|z, w, \eta) \propto \prod_{d=1}^{D}\prod_{n=1}^{N_d} p(w_{d,n}|\beta_k)^{z_{d,n}} p(\beta_d|\eta) \\
&\propto \prod_{d=1}^{D}\prod_{n=1}^{N_d}\prod_{g=1}^{V} \beta_{k,g}^{w_{d,n}^g z_{d,n}^k} \prod_{g=1}^{V} \beta_{k,g}^{\eta-1} \\
&= \prod_{g=1}^{V} \beta_{k,g}^{\eta-1+\sum_{d=1}^{D}\sum_{n=1}^{N_d} w_{d,n}^g z_{d,n}^k} \\
&= \mathrm{Dir}(\eta + \sum_{d=1}^{D}\sum_{n=1}^{N_d} w_{d,n}^g z_{d,n}^k)
\end{aligned}$$

- The conditional distribution of cell type (component) assignment $z_{d,n}$ is given as

$$\begin{aligned}
p(z_{d,n} = k|z_{-(d,n)}, \theta, \beta, w) &= p(z_{d,n}|\theta_{d,}, \beta, w_{d,n}) \\
&\propto p(\theta_d)p(z_{d,n} = k|\theta_d)p(w_{d,n}|\beta_k) \\
&= \theta_{d,k} p(w_{d,n}|\beta_k)
\end{aligned}$$

Gibbs Sampler exhibits a slow convergence rate. Moreover, in each iteration, if a gene expression β_k assigns a probability of 0 to a gene $w_{d,n}$, then it will have a posterior probability of 0 under $z_{d,n}$. Consequently, the information of $z_{d,n}$ will be lost in the next iteration of θ_d. To address these challenges, the Collapsed Gibbs Sampler was proposed[3][4]. The conditional probability of cell type assignment k is proportional to the joint probability of the assignment and the gene:

$$p(z_{d,n} = k|z_{-(d,n)}, w) \propto p(z_{d,n} = k, w_{d,n}|z_{-(d,n)}, w_{-(d,n)})$$

Given the cell type proportions and gene expressions, the joint distribution of a cell type assignment and genes is:

$$\begin{aligned}
p(z_{d,n} = k, w_{d,n}|\theta_d, \beta_{1:K}) &= p(z_{d,n} = k|\theta_d)p(w_{d,n}|\beta_{1:K}, z_{d,n} = k) \\
&= \theta_{d,k}\beta_{k,w_{d,n}}
\end{aligned}$$

Next, integrating out the cell type proportions θ_d and gene expression β_k yields an integrand independent of the other assignments and genes. For brevity, we use the shorthand notation $z_{d,n} = k$ as $z_{d,n}$:

$$\begin{aligned}
p(z_{d,n}|z_{-(d,n)}, w) &\propto p(z_{d,n}, w_{d,n}|z_{-(d,n)}, w_{-(d,n)}) \\
&\propto \int_{\beta_k}\int_{\theta_d} p(\theta_d, \beta_k, z_{d,n}, w_{d,n}|z_{-(d,n)}, w_{-(d,n)}) \\
&= \int_{\beta_k}\int_{\theta_d} p(z_{d,n}, w_{d,n}|\theta_d, \beta_k)p(\theta_d|z_{d,-n})p(\beta_k|z_{-(d,n)}, w_{-(d,n)}) \\
&= \int_{\beta_k}\int_{\theta_d} \theta_{d,k}\beta_{k,w_{d,n}} p(\theta_d|z_{d,-n})p(\beta_k|z_{-(d,n)}, w_{-(d,n)}) \\
&= \left(\int_{\theta_d} \theta_{d,k} p(\theta_d|z_{d,-n})\right)\left(\int_{\beta_k} \beta_{k,w_{d,n}} p(\beta_k|z_{-(d,n)}, w_{-(d,n)})\right)
\end{aligned}$$

Each of the above two terms represents the expectation of posterior Dirichlet distributions. Thus, the final algorithm is given as

$$p(z_{d,n} = k|z_{-(d,n)}, w) = \left(\frac{N_{(\cdot)dk}^{-(d,n)} + \alpha}{N_{(\cdot)d(\cdot)} + K\alpha}\right)\left(\frac{N_{i(\cdot)k}^{-(d,n)} + \eta}{N_{(\cdot)(\cdot)k}^{-(d,n)} + V\eta}\right) \tag{3}$$

where,

- N_{gdk}: Number of genes of type g in spot d assigned to topic k.
- $N_{gdk}^{-(d,n)}$: The count N_{gdk} excluding the contribution of gene $w_{d,n}$.

A new value for $z_{d,n}$ is sampled for each gene $w_{d,n}$ during every iteration of Gibbs sampling. The sampler runs for a burn-in period of 1500 iterations to allow it to reach convergence, after which θ_d and β_k are estimated from z as follows:

$$\theta_{d,k} = \frac{N_{(\cdot)dk} + \alpha}{N_{(\cdot)d(\cdot)} + K\alpha}$$

$$\beta_{k,g} = \frac{N_{g(\cdot)k} + \eta}{N_{(\cdot)(\cdot)k} + V\eta}$$

For our gwSPADE algorithm, we considered gene frequency weights to improve the deconvolution results. Specifically, equation (3) is modified by

$$\begin{aligned}
p(z_{d,n} = k|z_{-(d,n)}, w) &= \left(\frac{M_{(\cdot)dk}^{-(d,n)} + \alpha}{M_{(\cdot)d(\cdot)} + K\alpha}\right)\left(\frac{M_{g(\cdot)k}^{-(d,n)} + \eta}{M_{(\cdot)(\cdot)k}^{-(d,n)} + V\eta}\right) \\
&= \frac{\sum_{g=1}^{V} m(w_{d(\cdot)}^g)N_{gdk}^{-(d,n)} + \alpha}{\sum_{g=1}^{V} m(w_{d(\cdot)}^g)N_{gd(\cdot)}^{-(d,n)} + K\alpha} \cdot \frac{\sum_{d=1}^{D} m(w_{d(\cdot)}^g)N_{gdk}^{-(d,n)} + \eta}{\sum_{g=1}^{V}\sum_{d=1}^{D} m(w_{d(\cdot)}^g)N_{gdk}^{-(d,n)} + V\eta}.
\end{aligned} \tag{4}$$

where $w_{d,n}^g$ denotes that the nth gene in spot d is of gene type g, $m(w_{d,n}^g)$ is the weighting term of the corresponding gene type. Notice that, if all weights $m(\cdot) = 1$, this reduces to the standard LDA formulation in equation (3).

2 Model-based data simulation

For each β_k, $k = 1, 2, 3, 4$, we used 'gtools::rdirichlet()' to generate pseudo cell type expression profiles for 100 genes (g1, g2, $\cdots$, g100). To simulate cell type proportions θ, we applied 'gtools::rdirichlet(n_spots, rep(1/K, K))' to generate cell type proportions across all spots. Assuming an average total count of 1000 per spot, we simulated total counts using a Poisson distribution. For each spot, we first sampled the number of counts contributed by each cell type from a multinomial distribution parameterized by θ_d, and then sampled gene counts for each cell type from a multinomial distribution based on the corresponding β_k.

To evaluate the consistency of gene number selection in our application, we generated an additional simulation using pseudo cell type expression profiles comprising 10,000 genes across 8 cell types. High frequency genes were designed to be predominantly specific to particular cell types. We simulated the distribution of these cell types across 2,000 spots with random cell type assignments, each spot containing an average of 10,000 total counts. We then selected the top 500, 1,000, and 2,000 highly variable genes to perform the deconvolution and assess performance under different gene thresholds.

3 Real data pre-processing

3.1 MOB ST data

We obtained mouse olfactory bulb (MOB) datasets from the original publication, focusing on MOB replicate #8. Coarse clustering annotations were obtained from STdeconvolve. To ensure a direct comparison with STdeconvolve, We followed the same preprocessing steps. First, we removed genes with fewer than 100 reads detected across spots, and excluded spots with fewer than 100 total gene counts. This filtering process resulted in a cleaned dataset containing 260 spots and 7,365 genes. Next, we selected 255 overdispersed genes using the default generalized additive model (basis = 5) and applied multiple testing with an adjusted p-value < 0.05. We fit the model using integer values of K from 2 to 18 and selected $K = 7$, which minimized perplexity and resulted in fewer rare cell types with mean spot proportions below 5%.

3.2 10x Visium data

A 10x Visium dataset of a coronal section of the mouse cortex was obtained. We removed spots with fewer than 100 gene counts and genes with fewer than 100 total counts, resulting in a dataset with 2,702 pixels and 13,548 genes. To retain a diverse set of biologically significant genes, we only removed genes detected in fewer than 1% or in 100% of spots. From the remaining genes, we selected the top 1,000 most significant overdispersed genes using a generalized additive model (basis = 5) with an adjusted p-value< 0.05. We then fit gwSPADE-BDC with integer values of K ranging from 8 to 20 and selected $K = 13$ based on its lower perplexity and smaller number of 'rare' cell types, defined as those with mean spot proportions below 5%.

3.3 DBiT-seq data

We obtained the DBiT-seq dataset of an E11 mouse embryo lower body sample (GSM4364242_E11-1L) from the original publication[5]. After removing spots with fewer than 100 gene counts, genes with fewer than 100 total counts, and mitochondrial genes, we were left with a filtered dataset containing 1,831 spatial locations and 7,171 genes. We then performed feature selection, identifying the top 1,000 most significantly overdispersed genes using a generalized additive model (basis = 5)

with an adjusted p-value< 0.05. Genes were retained if they were detected in more than 1% but fewer than 100% of the spots. gwSPADE-BDC was fit with integer values of K ranging from 8 to 20, and $K = 13$ was selected based on its lower perplexity and a smaller number of 'rare' cell types (defined as those with mean spot proportions $< 5\%$). This value of K corresponds to the number of transcriptional clusters identified for this sample in the original publication.

3.4 PDAC ST data

We obtained the PDAC dataset from the original publication[6]. For comparison with CARDfree, we used the marker gene lists provided by CARDfree, resulting in 1,379 marker genes that were present in the count matrix. To ensure a fair comparison across different deconvolution methods, we standardized the analysis by implementing deconvolution on the same scaled count matrix with 428 spots and 1,379 genes, setting $K = 20$, corresponding to the number of cell types in the marker gene lists.

3.5 NT-PDAC ST data

We obtained the NT-PDAC ST dataset from one of the samples reported in Wu et al.[7]. The sample contained 3,885 spots and was first deconvolved using Cottrazm[8], a reference-based deconvolution method. Then, we focused on spots with the proportion of ductal cell type $> 60\%$ and used gwSPADE to identify sub-cell types. The $> 60\%$ ductal purity leads to 1,127 spots. We first removed 93 plasma genes and genes expressed in less than 1% but more than 100% of the spots. Then, we selected significantly overdispersed genes using a generalized additive model (basis = 5) with an adjusted p-value< 0.05, leading to a total of 748 genes included for further deconvolution analysis. We then fit gwSPADE-BDC with integer values of K ranging from 2 to 10 and selected $K = 5$ based on its lower perplexity and low number of 'rare' cell types defined as those with mean spot proportions below 5%.

4 Supplementary Figures

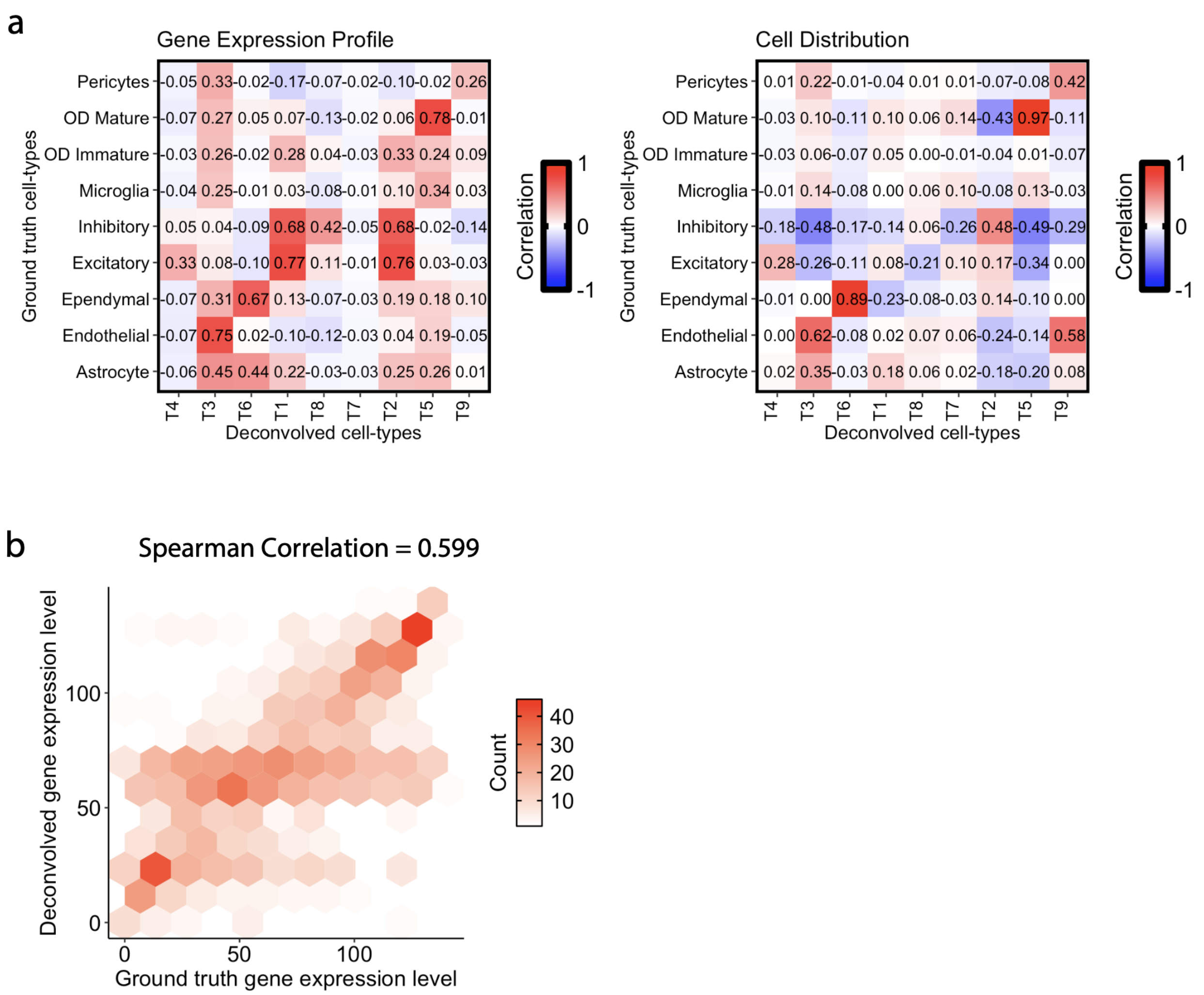

Figure S1: **SpiceMix in the MPOA data.** **a** (Left) Pearson's correlation between the transcriptional profiles of the 9 ground truth cell types in the MERFISH MPOA data and the corresponding 9 deconvolved cell types. (Right) Pearson's correlation between the simulated grid proportions of the 9 ground truth cell types and the 9 deconvolved cell types. **b** Gene ranking comparison based on expression levels in deconvolved cell type transcriptional profiles, relative to their rankings in the matched ground truth cell type transcriptional profiles, along with the corresponding Spearman's correlation coefficient (SCC)

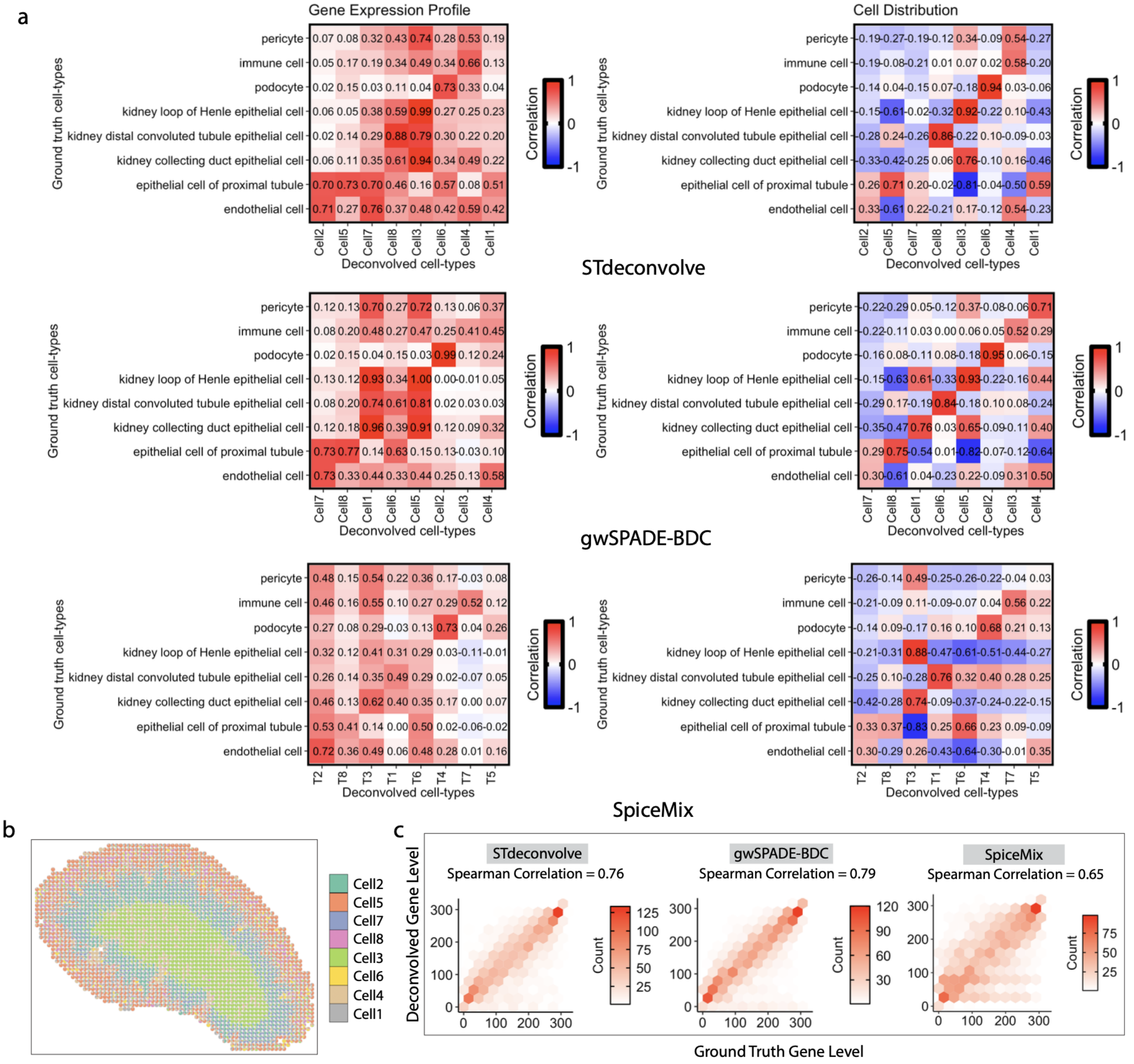

Figure S2: **Comparision between STdeconvove, SpiceMix and gwSPADE in the MK data.** **a** (Left) Pearson's correlation between the transcriptional profiles of the 8 ground truth cell types in the MERFISH MK data and the corresponding 8 deconvolved cell types. (Right) Pearson's correlation between the simulated grid proportions of the 8 ground truth cell types and the 8 deconvolved cell types. (Top: STdeconvolve; Middle: gwSPADE-BDC; Bottom: SpiceMix). **b** Predicted grid proportions of 8 deconvolved cell types from STdeconvolve. **c** Gene ranking comparison based on expression levels in deconvolved cell type transcriptional profiles, relative to their rankings in the matched ground truth cell type transcriptional profiles, along with the corresponding Spearman's correlation coefficient (SCC) (Left: STdeconvolve; Middle: gwSPADE-BDC; Right: SpiceMix

).

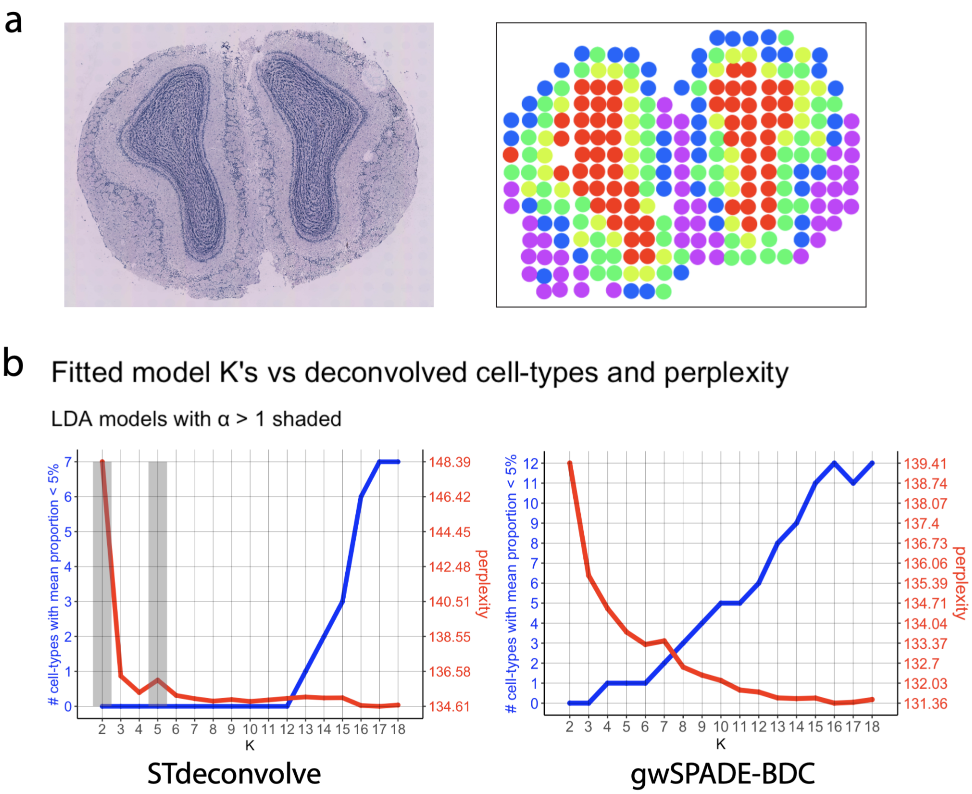

Figure S3: **Additional plots related to the MOB data.** **a** (Left) H&E-stained image of the mouse olfactory bulb (MOB) tissue section. (Right) Visualization of the corresponding MOB spots, colored by their transcriptional cluster memberships mapped to spatial locations. **b** Plot of perplexity and the # of cell types with mean proportional $< 5\%$ under different numbers of cell types K for STdeconvolve and gwSPADE-BDC.

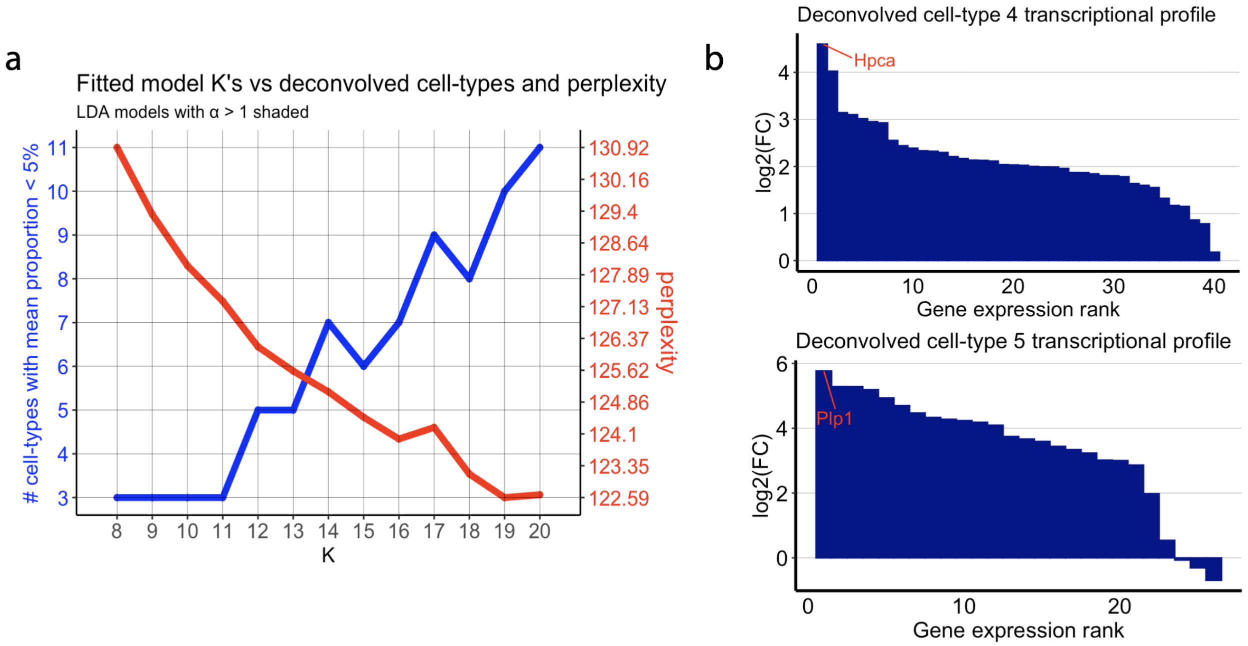

Figure S4: **Additional plots related to the 10× Visium mouse brain data. a** Plot of perplexity and the # of cell types with mean proportional < 5% under different numbers of cell types K for gwSPADE-BDC. **b** Log2 fold-change analysis of deconvolved gene transcriptional profiles for each deconvolved cell type compared to the mean deconvolved expression of the other 12 cell types (Top: for cell type 4; Bottom: for cell type 5.).

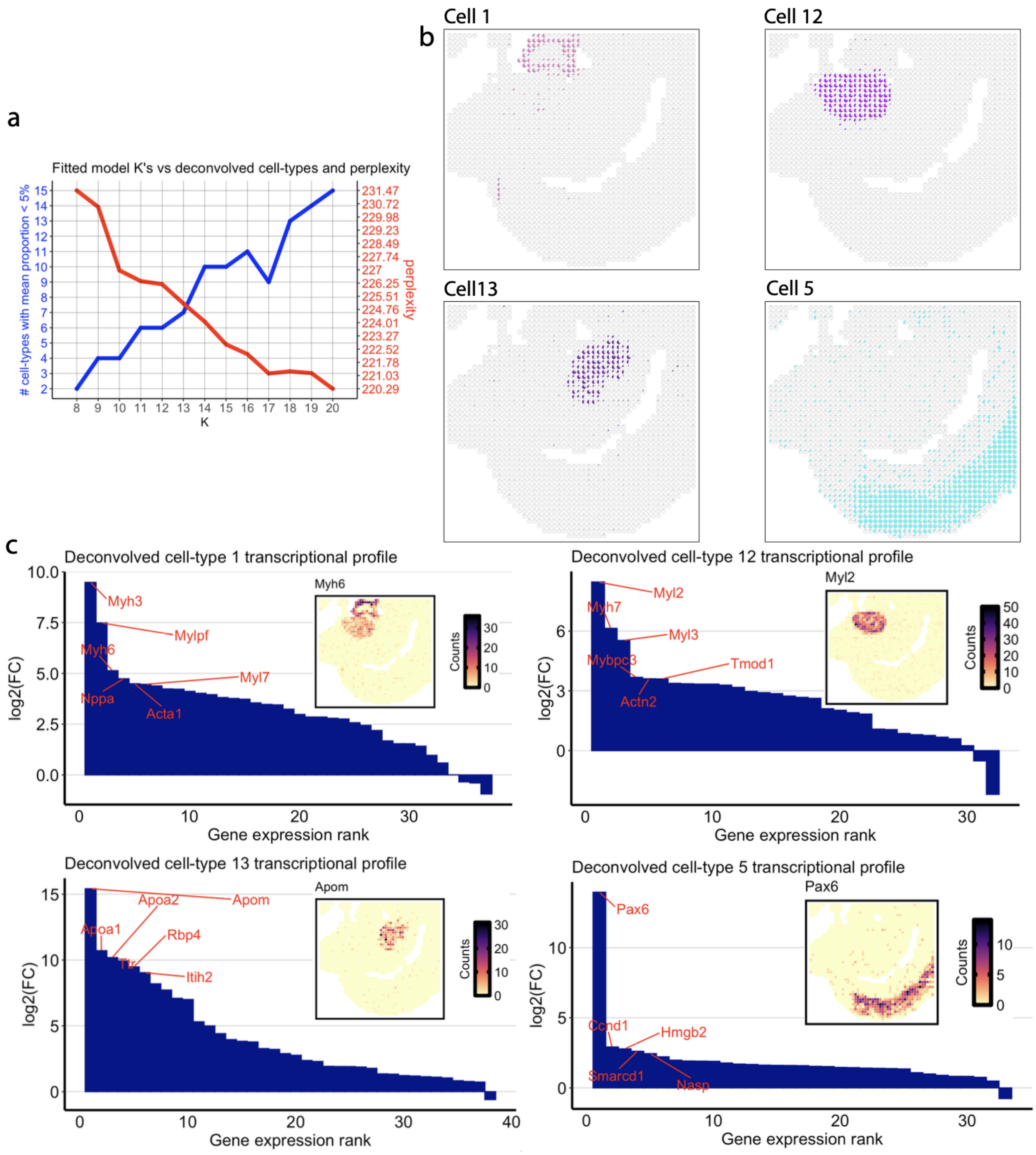

Figure S5: **Spots proportions and log2 fold of transcriptional profiles of the selected deconvolved cell-types in DBiT-seq data of the E11 mouse embryo lower tail section.** **a** The relationship between the number of cell types K and the number of "rare" cell types, along with perplexity scores for gwSPADE-BDC. **b** Visualization of the spot proportions for select deconvolved cell types, Cell1 (atrium), Cell12 (ventricle), Cell13 (fetal liver), and Cell5 (neural tube), corresponding to annotations from a previous publication[5]. **c** Log2 fold-change analysis of the deconvolved transcriptional profile of these cell types with respect to the mean expression of the other 12 deconvolved cell types. The expression of the top differentially expressed genes in each deconvolved transcriptional profile is visualized in the original tissue (inset).

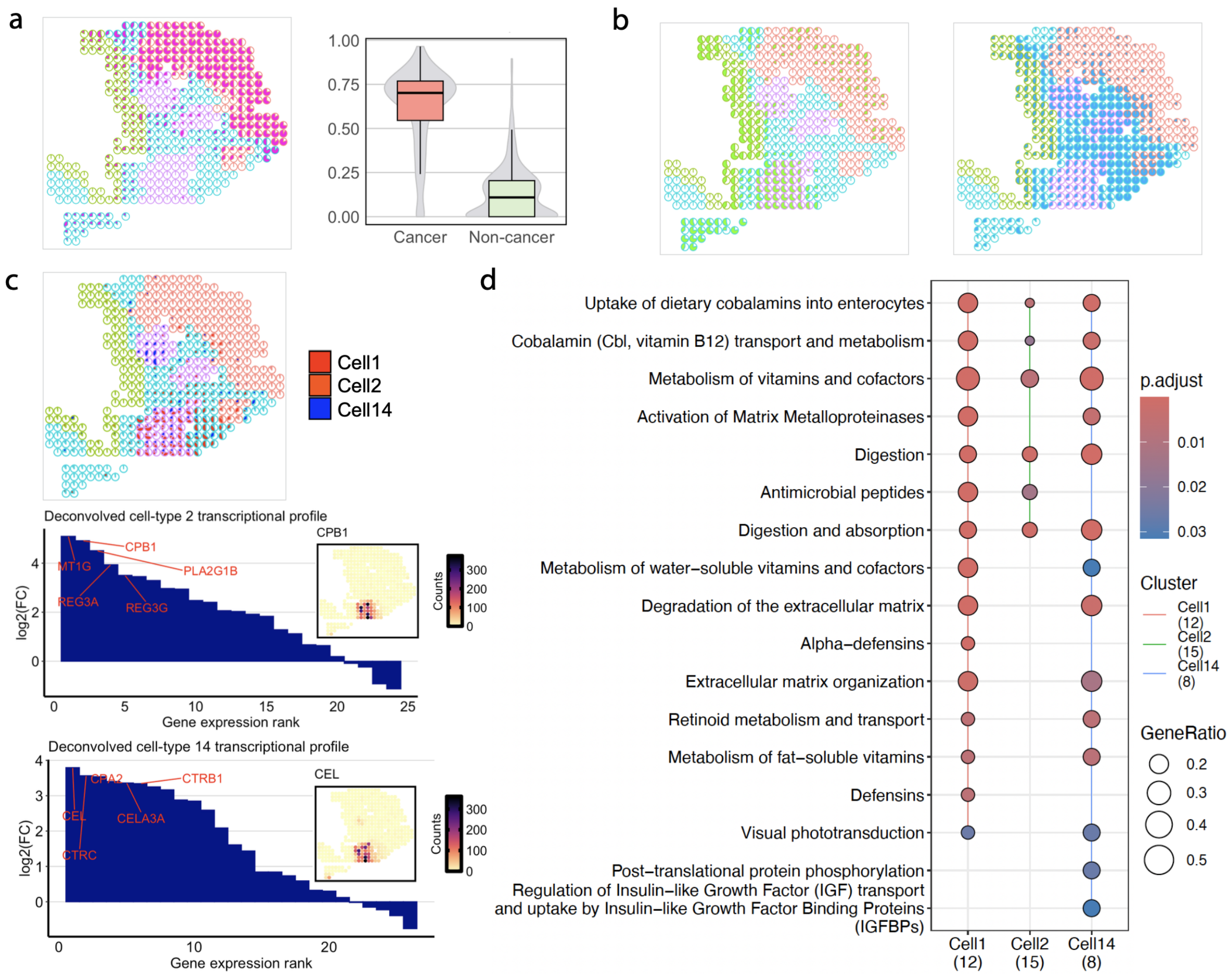

Figure S6: **SpiceMix deconvolution and Analysis of Acinar cells in the gwSPADE-BDC of the PDAC ST data.** **a** (Left) Highlights of the identified deconvolved cell types for tumors from SpiceMix. (Right) Comparison of cell-type proportions inferred by SpiceMix in cancer regions versus non-cancer regions. **b** Visualization of major deconvolved cell types in different regions. (Left) Ductal region. (Right) Stroma region. **c** (Top) Visualization of deconvolved acinar-adjacent cell types, Cell1, Cell2, and Cell14, showing their compositions across spatial spots. (Bottom) Log2 fold-change of Cell2 and Cell14 transcriptional profiles relative to the average expression of the other 19 deconvolved cell types, with visualization of the top differentially expressed genes in each deconvolved cell type. **d** Pathway enrichment analysis of top expressed genes from Cell1, Cell2, and Cell14 based on the Reactome pathway. The number below each cell type represents the number of overlapped genes between the top expressed genes of that cell type and all genes in the Reactome collection. The GeneRatio represents the ratio of the overlap size between top expressed genes of a specific cell type and a particular pathway gene set to the total overlap with all Reactome pathway members.

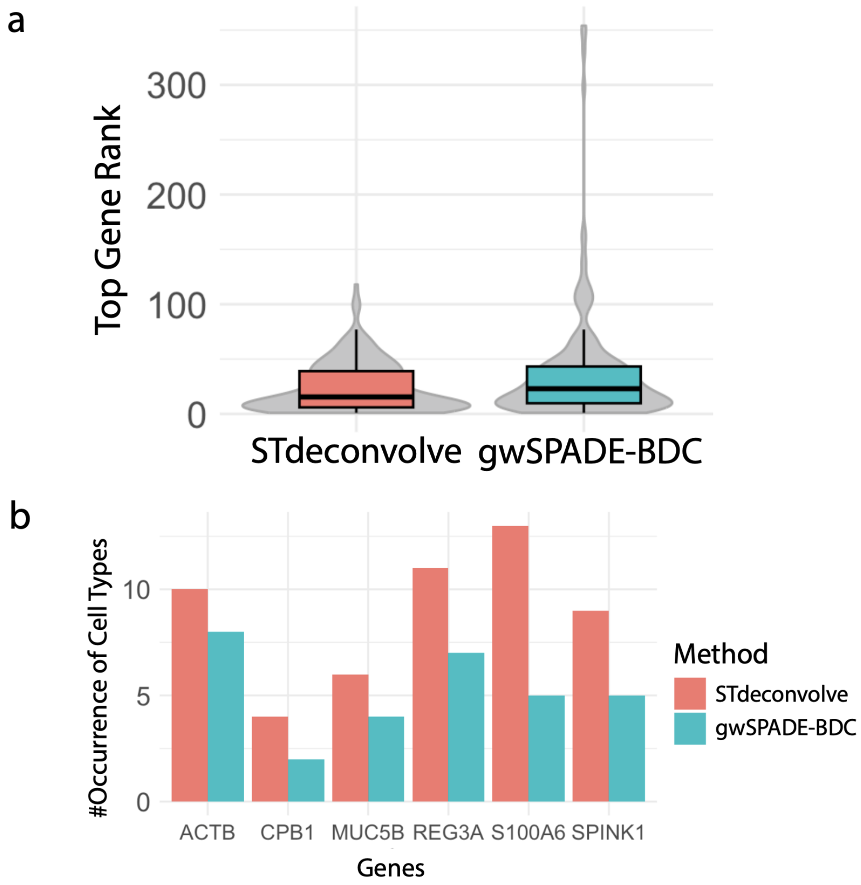

Figure S7: **Genes domination comparision between STdeconvolve and gwSPADE-BDC. a** Genes were ranked by their expressions in the entire count matrix, from highest (small rank values) to lowest (large rank values). The top 10 highly expressed genes for each deconvolved cell type were then selected to examine the rank distribution of these genes in STdeconvolve and gwSPADE-BDC. For STdeconvolve, genes with high expressions dominate the top 10 highly expressed gene list in different cell types, while for gwSPADE-BDC, some low expression genes also appear in the top 10 gene list due to upweighting, thus providing high discriminative power for improved deconvolution. **b** Comparison of the frequency of occurrence of high-frequency genes in the top 10 highly expressed gene list in the 20 deconvolved cell types with STdeconvolve and gwSPADE-BDC. For example, gene *ACTB* appeared in the top 10 gene list in 10 cell types with STdeconvolve, while it appeared in 8 cell types with gwSPADE, showcasing the downweighting effect by the BDC weighting scheme.

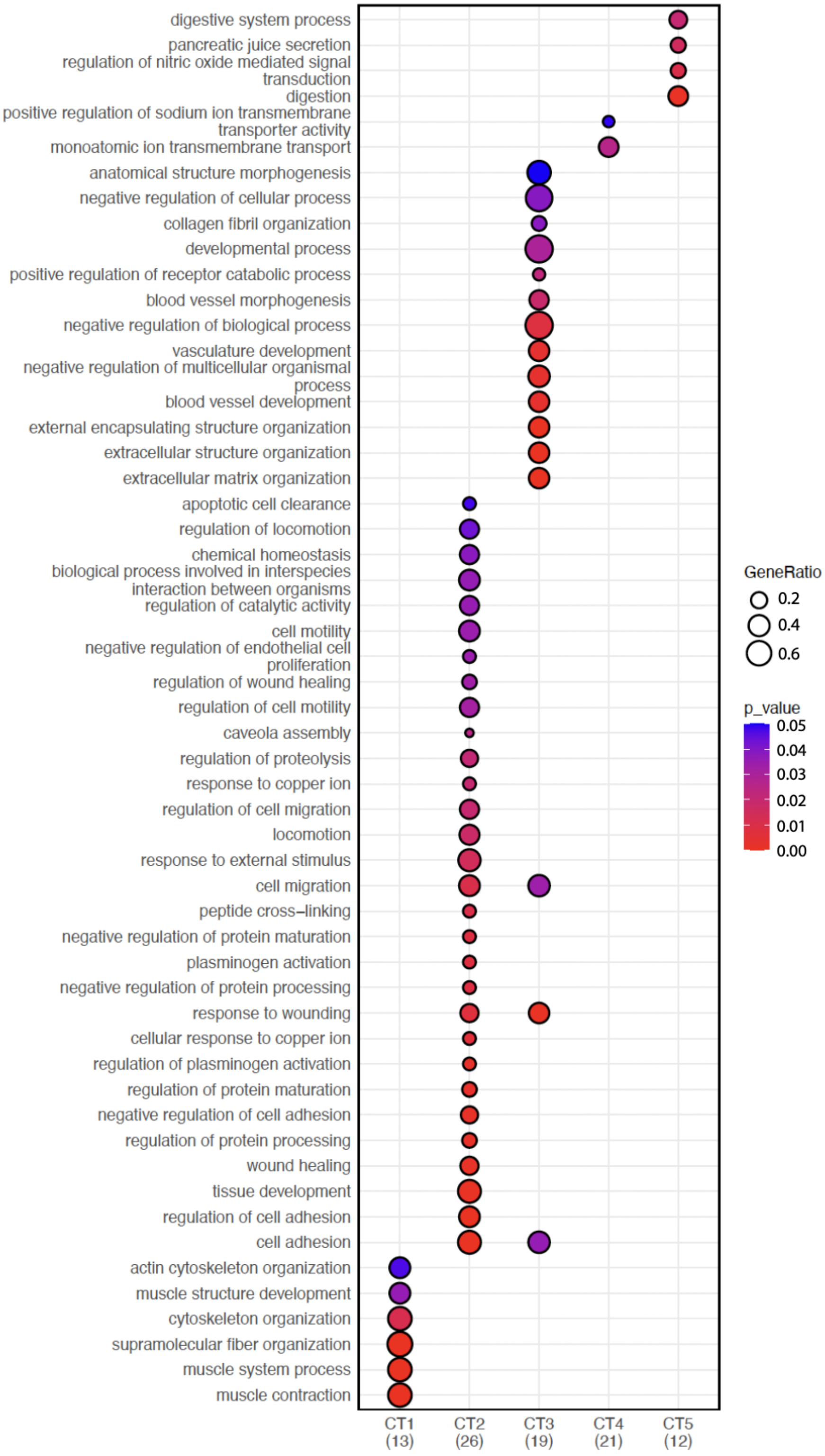

Figure S8: **GO term Biological Process (BP) enrichment.** Pathway enrichment analysis of unique top expressed genes from inferred sub-cell types based on the GO term BP. The number below each sub-cell type (denoted as CT) represents the number of unique genes in that sub-cell type overlapped with genes in that GO term pathway. The GeneRatio represents the proportion of genes in a specific GO term relative to the overlapped genes in that sub-cell type (the number below CT).

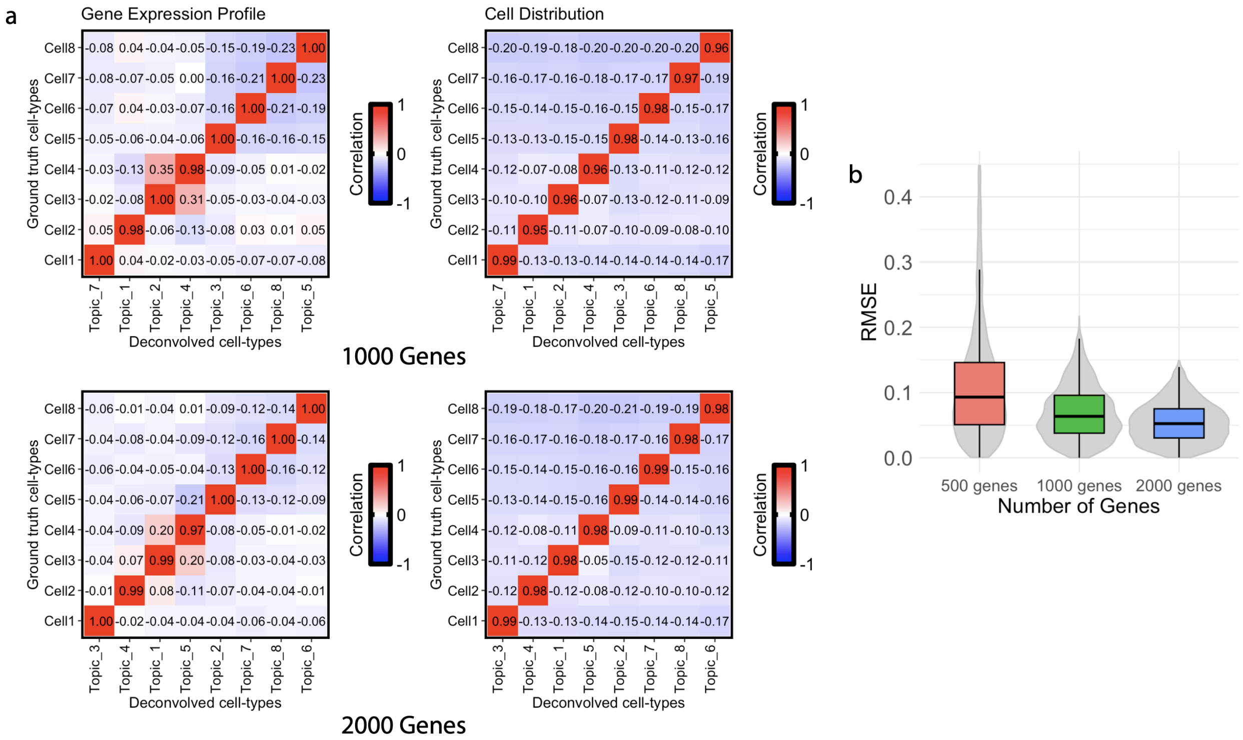

Figure S9: **Deconvolved Results Comparison among different number of HVGs. a** (Left) Pearson's correlation between the transcriptional profiles of the 8 ground truth cell types in simulated data and the corresponding 8 deconvolved cell types. (Right) Pearson's correlation between the simulated spot proportions of the 8 ground truth cell types and the 8 deconvolved cell types. (Top: 1000 genes; Bottom: 2000 genes) **b** Boxplot of RMSEs of the deconvolved cell-type proportions.

References

[1] D. M. Blei, A. Y. Ng, and M. I. Jordan, "Latent dirichlet allocation," *Journal of Machine Learning Research*, vol. 3, no. Jan, pp. 993–1022, 2003.

[2] B. F. Miller, F. Huang, L. Atta, A. Sahoo, and J. Fan, "Reference-free cell type deconvolution of multi-cellular pixel-resolution spatially resolved transcriptomics data," *Nature Communications*, vol. 13, no. 1, p. 2339, 2022.

[3] T. L. Griffiths and M. Steyvers, "Finding scientific topics," *Proceedings of the National Academy of Sciences*, vol. 101, no. suppl_1, pp. 5228–5235, 2004.

[4] D. M. Blei, "Mixed-membership models (and an introduction to variational inference)," *Course notes for Foundations of Graphical Models. Nov*, 2015.

[5] Y. Liu, M. Yang, Y. Deng, G. Su, A. Enninful, C. C. Guo, T. Tebaldi, D. Zhang, D. Kim, Z. Bai, *et al.*, "High-spatial-resolution multi-omics sequencing via deterministic barcoding in tissue," *Cell*, vol. 183, no. 6, pp. 1665–1681, 2020.

[6] R. Moncada, D. Barkley, F. Wagner, M. Chiodin, J. C. Devlin, M. Baron, C. H. Hajdu, D. M. Simeone, and I. Yanai, "Integrating microarray-based spatial transcriptomics and single-cell rna-seq reveals tissue architecture in pancreatic ductal adenocarcinomas," *Nature Biotechnology*, vol. 38, no. 3, pp. 333–342, 2020.

[7] Y. Wu, D. Sålas-Escabillas, A. Xie, H. K. Loveless, I.M., R. Shankar, M. George, A. Wombwell, J. M. Clark, D. Kwon, B. Chen, R. Francescone, D. B. Vendramini-Costa, B. Theisen, L. Huang, Y. Cui, H. C. Crawford, and N. G. Steele, "Spatial transcriptomics on a diverse pancreatic cancer cohort reveals black african americans display reduced classical and increased intermediate tumor subtype," *Manuscript.*

[8] Z. Xun, X. Ding, Y. Zhang, B. Zhang, S. Lai, D. Zou, J. Zheng, G. Chen, B. Su, L. Han, *et al.*, "Reconstruction of the tumor spatial microenvironment along the malignant-boundary-nonmalignant axis," *Nature Communications*, vol. 14, no. 1, p. 933, 2023.
